# Exploring genetic variation among Jordanian *Solanum lycopersicon* L. landraces and their performance under salt stress using SSR markers

**DOI:** 10.1186/s43141-022-00327-2

**Published:** 2022-03-11

**Authors:** Ibrahim M. Makhadmeh, Samar G. Thabet, Mohammed Ali, Basmah Alabbadi, Ammar Albalasmeh, Ahmad M. Alqudah

**Affiliations:** 1 Department of Plant Production, Faculty of Agriculture, Jordan University of Science and Technology, Irbid, 22110 Jordan; 2 Department of Botany, Faculty of Science, University of Fayoum, Fayoum, 63514 Egypt; 3 Egyptian Deserts Gene Bank, Desert Research Center, Department of Genetic Resources, Cairo, 11753 Egypt; 4 Department of Natural Resources and Environment, Faculty of Agriculture, Jordan University of Science and Technology, Irbid, 22110 Jordan; 5 Department of Agroecology, Aarhus University Flakkebjerg, 4200 Slagelse, Denmark

**Keywords:** SSRs, Tomato, Landrace, Genetic variation, Salinity, Minerals

## Abstract

**Background:**

Tomatoes (*Solanum lycopersicon* L.) are one of the main daily consumed vegetables in the human diet. Tomato has been classified as moderately sensitive to salinity at most stages of plant development, including seed germination, seedling (vegetative), and reproduction phases. In this study, we evaluated the performance and response of 39 tomato landraces from Jordan under salt stress conditions. Furthermore, the landraces were also genetically characterized using simple sequence repeat (SSR) markers.

**Results:**

The studied morphological-related traits at the seedling stage were highly varied among landraces of which the landrace number 24 (Jo970) showed the best performance with the highest salt tolerance. The total number of amplification products produced by five primers (LEaat002, LEaat006, LEaat008, LEga003, LEta019) was 346 alleles. Primer LEta 019 produced the highest number of alleles (134) and generated the highest degree of polymorphism (100%) among landraces in addition to primers (LEaat002, LEaat006, LEaat008). The lowest dissimilarity among landraces ranged from 0.04 between accessions 25 (Jo969) and 26 (Jo981) and the highest dissimilarity (1.45) was found between accessions 39 (Jo980) and both 3 (Jo960) and 23 (Jo978). The dendrogram showed two main clusters and separated 30 landraces from the rest 9 landraces. High genetic diversity was detected (0.998) based on the average polymorphism information. Therefore, the used SSRs in the current study provide new insights to reveal the genetic variation among thirty-nine Jordanian tomato landraces. According to functional annotations of the gene-associated SSRs in tomatoes, a few of SSR markers gene-associated markers, for example, LEaat002 and LEaat008 markers are related to MEIS1 Transcription factors genes (*Solyc07g007120* and *Solyc07g007120.2*). The LEaat006 is related to trypsin and protease inhibitor (Kunitz_legume) gene (*Solyc03g020010*). Also, the SSR LEga003 marker was related to the Carbonic anhydrase gene (*Solyc09g010970*).

**Conclusions:**

The genetic variation of tomato landraces could be used for considering salt tolerance improvement in tomato breeding programs.

**Supplementary Information:**

The online version contains supplementary material available at 10.1186/s43141-022-00327-2.

## Background

Salinity condition is a significant threat to plant growth performance, preventing plants from reaching their development and genetic potential. Every year, salt stress conditions destroy about 20% of irrigated agriculture worldwide [1]. By 2050, the majority of irrigated agricultural lands may become unfit for agriculture because of the high dissolved salts in such lands [1, 2]. Moreover, weathering of rocks or salt deposits through precipitation, irrigation with saline water, and poor cultural practices management all contribute to the increasing soil salinity in the world's arid and semiarid regions. Globally, high salinity usually affects approximately 20% of irrigated arable lands and 33% of cultivated lands, and such areas are expanding at a rate of 10% per year [3]. Salinity stress conditions can also disrupt and induce changes in plant physiological, biochemical, and morphological characteristics, resulting in yield and performance losses [4, 5]. Therefore, it is critical to take initiatives to improve the salinity stress tolerance of crop varieties, particularly tomatoes (*Solanum lycopersicum* L.), which rank first among all vegetables in terms of nutrition and economic importance [6].

Tomato is a nightshade family member that is grown as an annual crop for fresh and processed use [7]. This crop is also used as a model plant to study angiosperm physiological responses, molecular genetics, and genomics under several environmental stimuli [8]. Tomato landraces are valuable sources of genetic characteristics that can be included in tomato breeding programs. High phenotypic variations were reported in tomato landraces [9]. However, morphological differences in tomatoes do not usually reflect genetic differences as they are highly influenced by environmental factors [10].

Salt stress has a major impact on tomato production, causing reductions in the growth performance and yield of tomato plants [11]. There are significant differences in salt stress tolerance among tomato species [12]. Wild tomato species (*Lycopersicon pimpinellifolium*, L. *peruvianum*, *L. cheesmanii*, *L. hirsutum*, and *L. pennellii* [13] are more salt-tolerant than cultivated tomatoes (*L. lycopersicum*). The majority of commercial tomato cultivars are moderately sensitive to salt stress, which has an impact on seed germination as well as the plant developmental stages [14]. Salinity harmed root length, shoot and root biomass, plant height, and leaf area, and ultimately lead to yield losses in tomato plants [15]. The negative effects of salt stress on seedlings’ morphology were caused by a change in plant physiology, which included altered water and nutrient absorption, the hormonal level that can change root and shoot signals [16]. Plants have developed a ubiquitous mechanism to tolerate salt stress conditions which involves ion compartmentalization in cell vacuoles and accumulation of compatible organic solutes in the cytosol for tuning the osmotica and reducing the influence of oxidative damage [17, 18]. Therefore, according to Singh et al. [9], improvement of salt tolerance in tomatoes could be achieved through the involvement of classical breeding programs, advances in biotechnology, or agricultural practices.

The natural genetic variation in tomato has been investigated in several studies using molecular markers such as isozymes, restriction fragment length polymorphisms (RFLPs), oligonucleotide fingerprinting, random amplified polymorphic DNA (RAPD) [19], amplified fragment length polymorphism (AFLP) [20], and simple sequence repeats (SSRs) [21]. SSRs revealed little information caused by a lack of variability, which was attributed to the self-pollinating nature of modern tomato cultivars associated with their narrow genetic base [22]. SSRs also known as microsatellite markers are short (2–5 nucleotides) tandem DNA repeats that have core sequences of 1–5 bp. They vary in number and motif for all repeat units and are flanked by conserved DNA sequences, such as the sequence pattern found in all Eukaryotic genomes [23]. SSRs were detecting the difference between and within species and can be found anywhere in the genome (coding, non-coding region, and promoter regions). Each SSR locus has a distinct flanking sequence, and primers targeting specific flanking regions could be intended to generate an SSR marker [24]. SSRs are powerful molecular markers, simple, easy to use, and highly reproducible of polymorphism, and they are used for the study of genetic diversity [25]. SSR markers had been widely used to discover the genetic variation of many crop plants, for example, date palms, citrus, asparagus, mung bean, and Medicago [26–30]. Alvarez et al. [22] used 17 microsatellite loci to evaluate genetic diversity in the *Lycopersicon* genus and found a high level of polymorphism, as well as many alleles, and the cross-pollinating species have higher gene diversity compared to self-pollinating species. According to He et al. [31], 129 new SSR microsatellite markers were developed and characterized for *S. lycopersicum* L. Thus, SSR markers are becoming the preferred molecular marker to identify the natural variation in tomatoes.

Therefore, this study aimed to (i) evaluate Jordanian tomato landraces grown under rainfed conditions morphologically and at the molecular level using SSR markers; (ii) examine the influence of different levels of NaCl salt stress on plant performance at seedling and vegetative developmental stage of tomato landraces; (iii) predict the candidate genes that associated with our SSRs primers in tomato; (iv) determine the putative tissue expression pattern, subcellular localization, root cell types- and tissues specific of our target genes. In the current study, we discovered the genetic diversity among tomato landraces using SSR markers. The output demonstrated the importance of landraces for salt tolerance and improving tomato fruit quality. The combined data analyses provide new insights into the possibility of valuable trait introgression in target tomato varieties to overcome salinity in salt tolerance breeding programs.

## Methods

### Plant materials

In this study, 39 tomato landraces from the National Agricultural Research Center (NARC) Genebank representing Jordan's geographical distribution were used and described in Table 1.

**Table 1:** General information regarding the collection including accession code and number, region of location in Jordan and fruit shape of tomato landraces used in this study

Code	Accession No.	Region of Location	Fruit shape
1	111A	Kharja	rounded
2	111B	Kharja	rounded
3	960	Shatanah	Slightly Flattened
4	951	Al’al	Rounded
5	952	Al’al	Rounded
6	956	Hebras	Flattened
7	995	WadiMusa	Flattened
8	972	Rhaba	Rounded
9	973	Rhaba	Flattened
10	967A	Rhaba	Flattened
11	967B	Rhaba	Flattened
12	971A	Rhaba	Flattened
13	971B	Rhaba	Rounded
14	961	Ain Jannah	U-shape
15	979	Rhaba	Flattened
16	988	Ain AlBaida	Flattened
17	989	Ain AlBaida	Flattened
18	968	Rhaba	Rounded
19	958	Sakib	Flattened
20	974A	Rhaba	High round
21	974B	Rhaba	Slightly flattened
22	994A	Shoubak	Flattened
23	978	Rhaba	Slightly flattened
24	970	Rhaba	Rounded
25	969	Rhaba	Flattened
26	981	Afra	Flattened
27	991A	Ain AlBaida	Rounded
28	991B	Ain AlBaida	Rounded
29	964	Rhaba	Flattened
30	959	Anjara	Slightly flattened
31	976	Rhaba	Flattened
32	975	Rhaba	Flattened
33	963	Rhaba	Rounded
34	985	Ain AlBaida	Flattened
35	986	Ain AlBaida	Flattened
36	987	Ain AlBaida	Flattened
37	957	Hebras	Rounded
38	955	Qasfa	Slightly Flattened
39	980A	Afra	Rounded

### Effect of salinity on tomato landraces at seedling stage

Twenty tomato seeds of each landrace were grown in polystyrene trays which had been filled with peat and perlite (2:1). After sowing, each tray received 1 mL of liquid fertilizer (20:20:20) with 1 L of salt (control, 4 dS m^−1^, and 6 dS m^−1^) and was covered with a plastic sheet. A completely randomized design with three replicates was used in the experiment, which was conducted in a greenhouse.

### Vegetative measurements

#### Plant fresh and dry weights

After 45 days of growth, the plants were harvested. Five plants were randomly selected from each replicate for each treatment and used to evaluate root and shoot fresh weight (FW) as well as their dry weight (DW) for mineral analysis. The shoots and roots were kept in an oven at 65°C for 72 h to determine the dry weight.

$${\displaystyle \begin{array}{c}\mathrm{Reduction}\ \mathrm{in}\ \mathrm{shoot}/\mathrm{root}\ \mathrm{weight}\ \left(\%\right)=\left[\mathrm{shoot}/\mathrm{root}\ \mathrm{weight}\ \left(\mathrm{control}\right)-\mathrm{shoot}/\mathrm{root}\ \mathrm{weight}\ \left(\mathrm{salinity}\right)\ \mathrm{over}\ \mathrm{shoot}/\mathrm{root}\ \mathrm{weight}\ \left(\mathrm{control}\right)\right]\times 100\\ {}\mathrm{The}\ \mathrm{growth}\ \mathrm{rate}\ \mathrm{in}\ \mathrm{shoot}\ \mathrm{or}\ \mathrm{root}\ \mathrm{at}\ 45\ \mathrm{days}=\mathrm{shoot}\ \mathrm{or}\ \mathrm{root}\ \mathrm{fresh}\ \mathrm{weight}/45\ \mathrm{days}\\ {}\mathrm{Reduction}\ \mathrm{of}\ \mathrm{shoot}/\mathrm{root}\ \mathrm{growth}\ \mathrm{rate}\ \left(\%\right)=\left[\mathrm{shoot}/\mathrm{root}\ \mathrm{growth}\ \mathrm{rate}\ \left(\mathrm{control}\right)-\mathrm{shoot}/\mathrm{root}\ \mathrm{growth}\ \mathrm{rate}\ \left(\mathrm{salinity}\right)\ \mathrm{over}\ \mathrm{shoot}/\mathrm{root}\ \mathrm{growth}\ \mathrm{rate}\ \left(\mathrm{control}\right)\right]\times 100\end{array}}$$

#### Proline determination

We followed the procedure described by Bates et al. [32] to measure the proline content (PRO) using the leaves. Briefly, 400 mL of the mixture (1.25 g ninhydrin, 20 mL phosphoric acid, and 30 mL glacial acetic acid) was mixed with a 200-mL supernatant aliquot and heated in sealed test tubes at 100 °C for 1 h. Then, the mixture was cooled, and 4 mL of toluene was added. The absorbance was measured at 520 nm and the data was recorded as mmol g^−1^ DW.

#### Mineral analysis

Oven dry materials (shoots and roots) were prepared for ion analysis. Samples (0.51 g) were ashed at 550 °C for 5 h using Thermolyne muffle Furnace. Wet ashing was done by adding 10 mL of 2 N HCl and heat for 10 min at 70 °C. The extract was filtered into 50 mL volumetric flasks and deionized water was added to bring the volume to 50 mL. To determine Cl, 5 drops of potassium chromate were added to 5 mL of the solution. Then the solution was titrated to an endpoint of light brown color using 0.05 N AgNO_3_. Mg, Ca, K, and Na contents were determined using Varian Spectra AA 200 Atomic absorption. Adequate standards were made for each test. A UV spectrophotometer was used for P and Kjeldahl Nitrogen use for N determination [33].

#### Statistical analysis

The Genstat program was used to perform statistical analysis of variance (ANOVA) and the significant differences among landraces were detected at a probability level of *P* ≤ 0.05. Means were separated according to LSD (0.05). *T* test was used to compare mineral mean. Mean values of proline and minerals contents were analyzed using Statistical Package for Social Studies (SPSS). Dissimilarity matrix and dendrogram were constructed to evaluate the variation of proline and mineral contents among tomato landraces.

#### DNA extraction and simple sequence repeat (SSR) assays

The micro CTAB method was used to extract total DNA from 0.35 g of mature leaves [34]. The DNA was quantified using an ND-1000 spectrophotometer (Nanodrop Technologies, USA). We also measured the DNA quality with the absorbance between 260 and 280 nm wavelengths (A260/A280) according to Weising et al. (1995). Our research created seventeen SSR primers for tomato DNA fingerprinting. These SSR primers sequences were obtained from He et al. [31]. After an initial screening of seventeen SSR primers, amplification products of five primers were selected for further analysis (LEaat002, LEaat006, LEaat008, LEga003, and LEta019). The SSR name was prefixed with LE standing for *S. lycopersicum* and these primers are shown in Table 2. Therefore, these primers were used in amplification reactions to determine genetic variation among tomato landraces.

**Table 2:** The simple sequence repeat marker (SSR), their locus name, flanking primer sequence, melting temperature, and fragment sizes of the polymerase chain reaction (PCR) products

SSR name	Locus	Forward primer 5′-3′	Reverse primer 5′-3′	Tm°C	Expected Allele size (bp)
LEaat002	cLES403	gcgaagaagatgagtcta gag cat ag	ctctct ccc atgagttctcctctt c	53.55	110
LEaat006	cLET1M11	gcc acg tag tca tga tat aca tag	gcc tcg gac aat gaa ttg	45.9	175
LEaat008	THox1	gag tca aca gca tag tggaggagg	cgtcgcaattct cag gcatg	51.35	180
LEga003	ND	ttcggtttattctgccaa cc	gcctgtaggattttcgcc ta	45.65	245
LEta019	Lemsrepr	tgtagataacttcctagcgacaat c	acggacggatgg aca aat g	47.65	300

#### PCR amplification and product electrophoresis

All SSR markers were subjected to polymerase chain reaction (PCR) optimization. During the primer testing, a subset of the total number of plants was used for PCR reaction. The PCR amplification reaction was carried out using five SSR primers pairs by the Perkin Elmer GeneAmp PCR system 9600 (PE Biosystems) or the TECHNE Genius thermal cycler (Techne Ltd., UK) (Table 2). The DNA from the 39 tagged tomato samples were finger-printed using SSR markers in a 10-μL reaction volume of the master mix which consisted of 25 ng DNA, 1× polymerase buffers [100 mM Tris HCL (PH 8), 15 mM MgCl_2_, 500 mM KCL, and 0.1% Difco Gelatin], 0.3 μM dNTPs, 1 unit of Taq polymerase (Promega, Madison, USA), 1.5 mM MgCl_2_, 0.03 μM from the forward and reverse primers with H_2_O was added to make the final volume. The PCR amplification is described as denaturation at 94 °C with 1 cycle for 2 min, followed by 33 cycles of amplification for 25 s denaturing at 94 °C, a 25-s annealing at the Tm (Tm varies for the individual primers), and a final elongation cycle at 68 °C for 5 min. After PCR amplification, the products were separated on 3% agarose gel (Metaphore Agarose) at 100 V for 1 h in TBE (Trisborateethelediamineteraacetic acid) (1×) using a gel electrophoretic (MS Major Science, UK) and BIO-RAD (Criterion TM cassettes), and loading dye was added into PCR products. A 5-μl of 100 bp DNA ladder (gene rule) was used as a reference in the PCR amplified products to find out the specific band size. Finally, DNA fragments were detected under UV-light using a gel documentation system (model Vilber Lourmat, IP-010-SD, France).

#### Data scoring and analysis

The gel of primers was analyzed through scoring the bands and coded by 0 and 1 absent/present bands, respectively. The genetic dissimilarity method of SSR markers was analyzed following Nei [35]. The unweighted pair group method with arithmetic average (UPGMA) cluster analysis applying the NTSYS-pc program version 2.1 by Rohlf (Exeter, Software, New York) were calculated. The polymorphism information content (PIC) of the SSRs was following Saal, Wricke [36]. The following equation was used:

$$\mathrm{PIC}=1-\overset{k}{\Sigma}{Pi}^2$$

Where *Pi* = frequency of the *i*th allele. *K* = total number of different alleles for that locus

#### Functional assignments for gene-associated SSRs in tomato

The sequence of SSRs markers was compared using various databases, such as Phytozome, National Center for Biotechnology Information (NCBI) Genbank, InterPro, and KEGG databases to predict the candidate genes associated with our SSRs primers in tomatoes. For potential functions of these genes, Phytozome v13 was used to obtain the annotations by KOG (Eukaryotic Orthologous Groups), KEGG (Kyoto Encyclopedia of Genes and Genomes), ENZYME, Pathway, and the InterPro family of protein analysis (classification of protein families) tools [37, 38]. In Phytozome we made the blast sequence against five tomato genomics such as *S. lycopersicum* ITAG2.4, *S. lycopersicum* ITAG3.2, *S. lycopersicum* ITAG4.0, *S. lycopersicum* v4.03, and *S. lycopersicum* v6.1. In context, we used SSRs primers sequence as a query to search in NCBI using the BLASTN tool.

#### Putative tissue expression pattern, subcellular localization, root cell types, and tissues specific of our target genes

Putative cell organs- and tissue-specific expression profile of *Solyc07g007120*, *Solyc03g020010*, *Solyc09g010970*, and *Solyc09g042380* genes from nineteen cell organs- and tissue-specific (such as; epidermis, collenchyma, vascular, parenchyma, endodermis, unopened flower bud, fully opened flower, leaves, root, 1 cm fruit, 2 cm fruit, 3 cm fruit, mature green fruit, breaker fruit, breaker fruit + 10, pimpinellifolium immature green fruit, pimpinellifolium breaker fruit, pimpinellifolium breaker + 5 fruit and pimpinellifolium leaf) were extracted based on *S. lycopersicum* transcript expression database. Expression profiles were built using the tomato plant Electronic Fluorescent Pictograph Browsers (Tomato eFP browsers (http://bar.utoronto.ca/eplant_tomato/). The bar represents the expression scale (the more intense the red color, the more gene expression). Moreover, the putative subcellular localizations of our previous gene from *S. lycopersicum* were examined based on tomato protein localization at different fourteen cell organs (such as cell plate, cytoskeleton, cytosol, endoplasmic reticulum, extracellular, golgi, mitochondrion, nucleus, peroxisome, plasma, membrane, plastid, and vacuole) to recognize possible synthesis sites using the tomato Cell eFP browsers (Tomato eFP browsers (http://bar.utoronto.ca/eplant_tomato/). Furthermore, the putative root cell types- and tissues-specific of our previous gene were examined at different root cell types- and tissues-specific under various promoter toolboxes, such as AtWER, SIPEP, AtPEP, SICO2, SISCR, SISHR, AtS32, AtS18, SIWOX5, SIRPL11C, and 35S promoters to determine the putative function of our genes at specific root cell types and their adaptive potential in distinct environments. The bar represents the expression scale (the more intense the red color, the more gene expression) http://bar.utoronto.ca/eplant_tomato/.

## Results

### Effect of salinity on tomato landraces seedlings growth

**Fig. 1:**
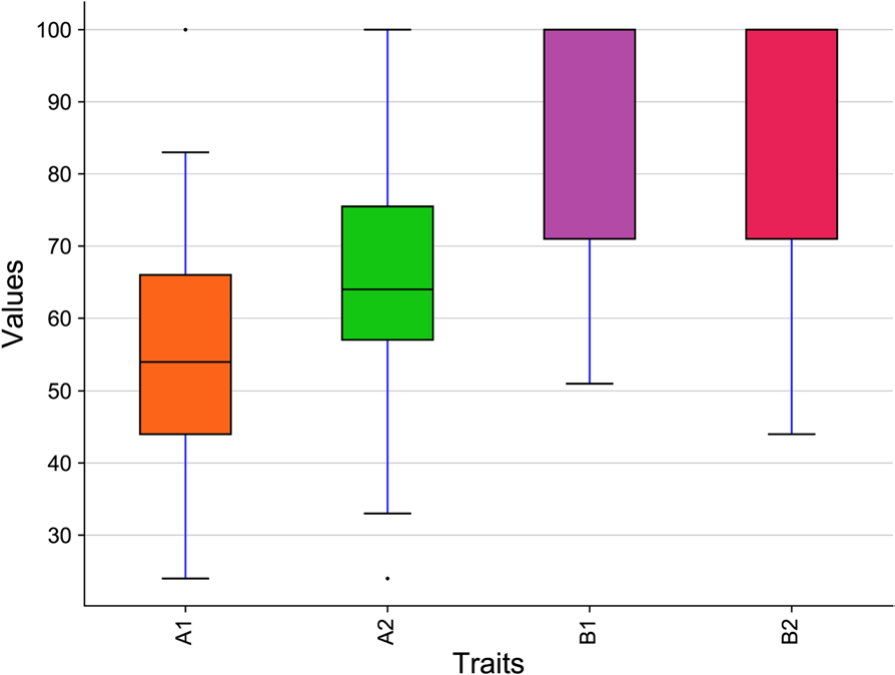
Box plots indicate the relative reduction of growth rate under both salinity levels of tomato landraces: (A1) relative reduction of shoot growth rate at 4 dS m−1, (A2) relative reduction of root growth rate at 4 dS m−1, (B1) relative reduction of shoot growth rate at 6 dS m−1, and (B2) relative reduction of root growth rate at 6 dS m−1

Shoot and root growth rates of the tested tomato landraces decreased significantly with increasing salinity when compared to the control (Fig. 1 and Table S1). For shoot fresh weight, the highest reduction at 4 dS m^−1^ and 6 dS m^−1^ salinity levels was observed for accession 1 (Jo111A) and accession 37 (Jo957) by 69% and 76%, respectively, whereas the lowest reduction was detected for accession 21 (Jo974A) and accession 24 (Jo970) by 22% and 51%, respectively (Table S2 and Table S3).

**Fig. 2:**
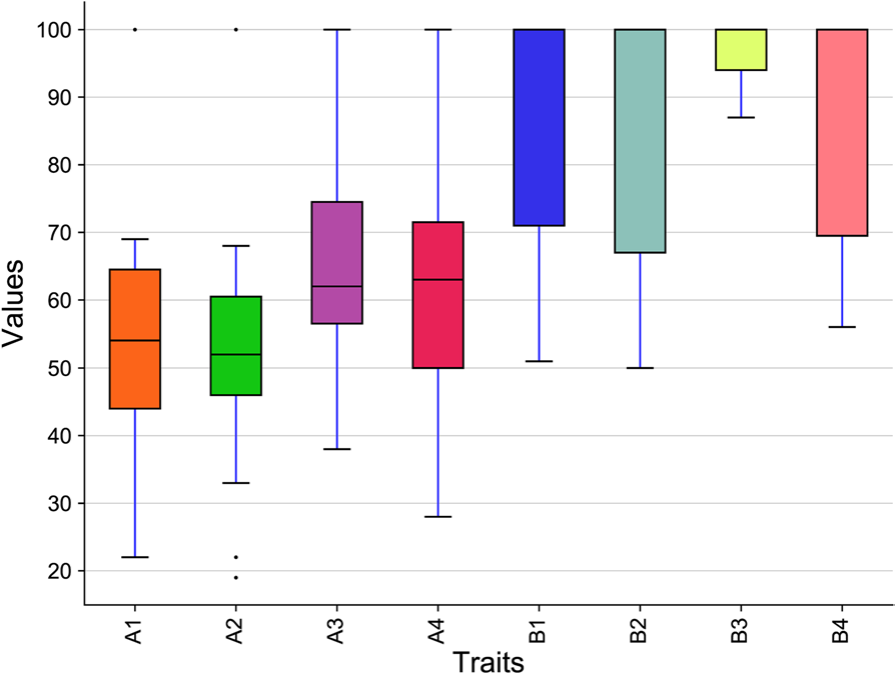
Box plots indicate the relative reduction of seedling related-traits under both salinity levels of tomato landraces: (A1) relative reduction of shoot fresh weight at 4 dS m−1, (A2) relative reduction of shoot dry weight at 4 dS m−1, (A3) relative reduction of root fresh weight at 4 dS m−1, (A4) relative reduction of root dry weight at 4 dS m−1, (B1) relative reduction of shoot fresh weight at 6 dS m−1, (B2) relative reduction of shoot dry weight at 6 dS m−1, (B3) relative reduction of root fresh weight at 6 dS m−1, and (B4) relative reduction of root dry weight at 6 dS m−1

At 4 dS m^−1^ and 6 dS m^−1^ salinity levels, the highest reduction in root fresh weight was recorded for accession 25 (Jo969) and accession 33 (Jo963) by 90% and 97%, respectively, while the lowest reduction was observed for accession 36 (Jo987) and accession 24 (Jo 970) by 38% and 87%, respectively (Fig. 2, Table S4 and Table S5). The highest reduction in shoot dry weight was recorded for accession 1 (Jo111A) by 19% and accession 37 (Jo957) by 76% at salinity levels 4 dS m^−1^ and 6 dS m^−1^, respectively, while the lowest reduction was observed for accession 21 (Jo974A) by 19% and accession 39 (Jo980A) by 50%, respectively (Table S6 and Table S7). For root dry weight, the highest reduction was 83% and 80% for accession 29 (Jo964) and accession 36 (Jo987) at salinity levels 4 dS m^−1^ and 6 dS m^−1^, respectively, while the lowest reduction was detected for accession 23 (Jo978) by 28% and accession 24 (Jo970) by 56%, respectively ((Table S8 and Table S9)). At 4 dS m^−1^ and 6 dS m^−1^ salinity levels, shoot growth rate showed the highest reduction (83%) for accession 29 (Jo 964) and (76%) for accession 37 (Jo 957), respectively, while the lowest reduction was noted for accession 19 (Jo 958) and accession 24 (Jo 970) by 24% and 51%, respectively (Table S2 and Table S10). Furthermore, root growth rate showed the highest reduction in accession 28 (Jo 991B) and accession 33 (Jo963) by 84% and 83%, respectively, while the lowest reduction was observed for accession 21 (Jo 974A) by 24% and accession 23 (Jo978) by 44%, respectively (Table S11 and Table S12).

Proline content from the leaves as mmol g^−1^ DW was varied between salinity level and landraces. Significant differences were found in proline content among landraces at salinity levels 4 and 6 dS m^−1^ (Table S13 and Table S14). The highest proline content was 195.8 at 4 dS m^−1^ compared with 3.1 under control was in accession 14 (Jo961), while the lowest proline content value under 4 dS m^−1^ treatment was 12.2 for accession 27 (Jo991A). At 6 dS m^−1^ salinity level, the highest proline content value was 142.3, 141.5, and 115.7 in accessions 24 (Jo970), 33 (Jo963), and 21 (Jo974A), respectively, whereas the lowest value was 50.6 in accession 39 (Jo980A) (Table S13 and Table S14).

### Mineral analysis

The average mineral content in shoots and roots of the 34 tomato accessions grown at 4 dS m^−1^ salinity level is presented in Table S15. The data revealed that Na^+^ content increased significantly with increasing salinity levels. Shoot and root average for Na^+^ contents in tomato landraces were higher under saline conditions than control. The average Na^+^ content in the shoot (3.90) was higher as compared to the root (2.24) at 4 dS m^−1^ salinity level for the 34 landraces (Table S15).

Mean values of Cl^-^ content in shoot and root were higher at 4 dS m^−1^ than control. Cl^-^ average content was higher in the shoot (9.9) than in root (3.97) under 4 dS m^−1^. The average K^+^ content in shoot and root was significantly decreased and shoot had higher content (4.8) than in root (1.71) for the tested 34 landraces. The average content of Ca in shoot and root was not significantly affected by salinity. The average Ca content in the shoot was 1.7 and in root was 0.98 (Table S15). In the shoot, data revealed that N, P, and Mg were significantly affected by the saline condition and there was an increase in N ( 4.2), while the average content of P and Mg were decreased by (0.7, 1), respectively (Table S15).

The average mineral content in shoots and roots of the 14 tomato accessions grown at 6 dS m^−1^ salinity level is presented in (Table S16). Increasing salinity up to 6 dS m^−1^ was not significantly affect N content (3.8), and Mg average content (0.9) as compared to 4 dS m^−1^. The average P, K, and Ca content in the shoot was significantly decreased as salinity increased, while the average content of K^+^ and Ca in root wasn’t significantly affected by increased salinity. Na^+^ and Cl^-^ content were significantly increased with increasing salinity in shoot and root. The average content of Na and Cl was higher in shoots than in roots (Table S17–Table S20).

### Cluster analysis of proline and mineral contents

The relationship among tomato landraces to proline and mineral contents was studied using cluster analysis. Distance between clusters is analyzed and reported as a dendrogram of dissimilarity to assess the relation among tomato landraces at two salinity levels (4 and 6 dS m^−1^). Proline content at 4 dS m^−1^ showed that the accessions were divided into two main clusters at the highest level of hierarchy (Figure S1), except for accession 14 (Jo961) which was separated in its group. The first main cluster grouped the accessions; 1 (Jo111A), 2 (Jo111B), 11 (Jo967B), 17 (Jo989), 26 (Jo981), and 33 (Jo963) while the remaining accessions were grouped in the second cluster. Ranging in similarity from 1.2 to 60.6 among accessions grouped in the first cluster and from 10 to 65 among accessions in the second group. The most divergent accession was 14 (Jo961) which form a separate cluster. At 6 dS m^−1^, two main clusters (Figure S2) were formed grouped accessions 24 (Jo970), 33 (Jo963), 21 (Jo974B) in the first main cluster and the remained accession grouped in the second cluster and were further subdivided into two sub-clusters. The dissimilarity coefficient ranged from 9 to 28 among accession in the first main cluster and from 2.8 to 43 among accessions in the second cluster.

For shoot mineral content at 4 dS m^−1^, the highest level of hierarchy in the dendrogram (Figure S3) accessions were grouped into two main clusters and were also sub-divided into two main sub-clusters. The first main cluster grouped 15 accessions out of 34 tested. The coefficient of dissimilarities ranged in this main group from 1.2 to 22.4 and from 1.1 to 14.1 in the second main cluster. The lowest distance was recorded between accession 14 (Jo961), 15 (Jo979) in the first cluster, while between accessions 36 (Jo987), 37 (Jo957) in the second cluster, and the highest distance was recorded among accessions 1 (Jo111A) and 39 (Jo980A).

At 6 dS m^−1^, the accessions were grouped in two main clusters at the highest level of hierarchy in the dendrogram (Figure S4). Accessions were grouped into two main clusters where accessions 21 (Jo974B), 23 (Jo978), 24 (Jo970), and 25 (Jo969) grouped in the first main cluster ranged in dissimilarity from 2 among accessions 21 (Jo974B) and 23 (Jo978) to 4.3 between accessions 21 (Jo974B) and 25 (Jo969). However, in the second main cluster, the remained accessions were further sub-divided into two main sub-clusters ranging in dissimilarity coefficient from 1.1 between accessions 36 (Jo987), 37 (Jo957) to 10.1 between accession, 29 (Jo964), and 39 (Jo980A).

For root mineral content at 4 dS m^-1^, the accessions were grouped in two main clusters at the highest level of hierarchy in the dendrogram, accessions ranged in dissimilarity from 1.1 among accessions 13 (Jo971B) and 14 (Jo961) to 14.2 among accessions 1 (Jo111A) and 15 (Jo979) in the first main cluster. In the second main cluster, accessions 18 (Jo968) and 19 (Jo958) were the closest with a 1.2 dissimilarity level, and accessions 17 (Jo989) and 39 (Jo980A), were the most diverse with a 22.0 dissimilarity coefficient. At 6 dS m^-1^, the accessions were grouped in two main clusters, accessions 21 (Jo974B), 25 (Jo969), 23 (Jo978), and 24 (Jo970) were grouped in the first cluster ranged in dissimilarity from 1.5 between accessions 23 (Jo978) and 24 (Jo970) to 7.3 between 1 (Jo111A) and 25 (Jo969), while the remained accessions were grouped in the second cluster with a range of dissimilarity from 1.1 between accession 33 (Jo963) and 34 (Jo985) to 10.4 between accession 29 (Jo964) and 39 (Jo980A), (Figure S5).

###  DNA quantification

The high intensities of DNA bands shown in Figure S6 indicated the high molecular weight of DNA with high purity. DNA concentration was also determined by using a spectrophotometer for all tomato landraces. The DNA concentration ranged from 300 to 900 ng with ratios ranging from 1.6 to 1.9. The DNA was diluted to several concentrations 50, 30, 25, 20, 15, 10, and 5 ng/μl. The 25 ng/μl DNA concentration produced the best reproducible pattern in amplification reactions.

### Allelic variation and SSR characterization

**Fig. 3:**
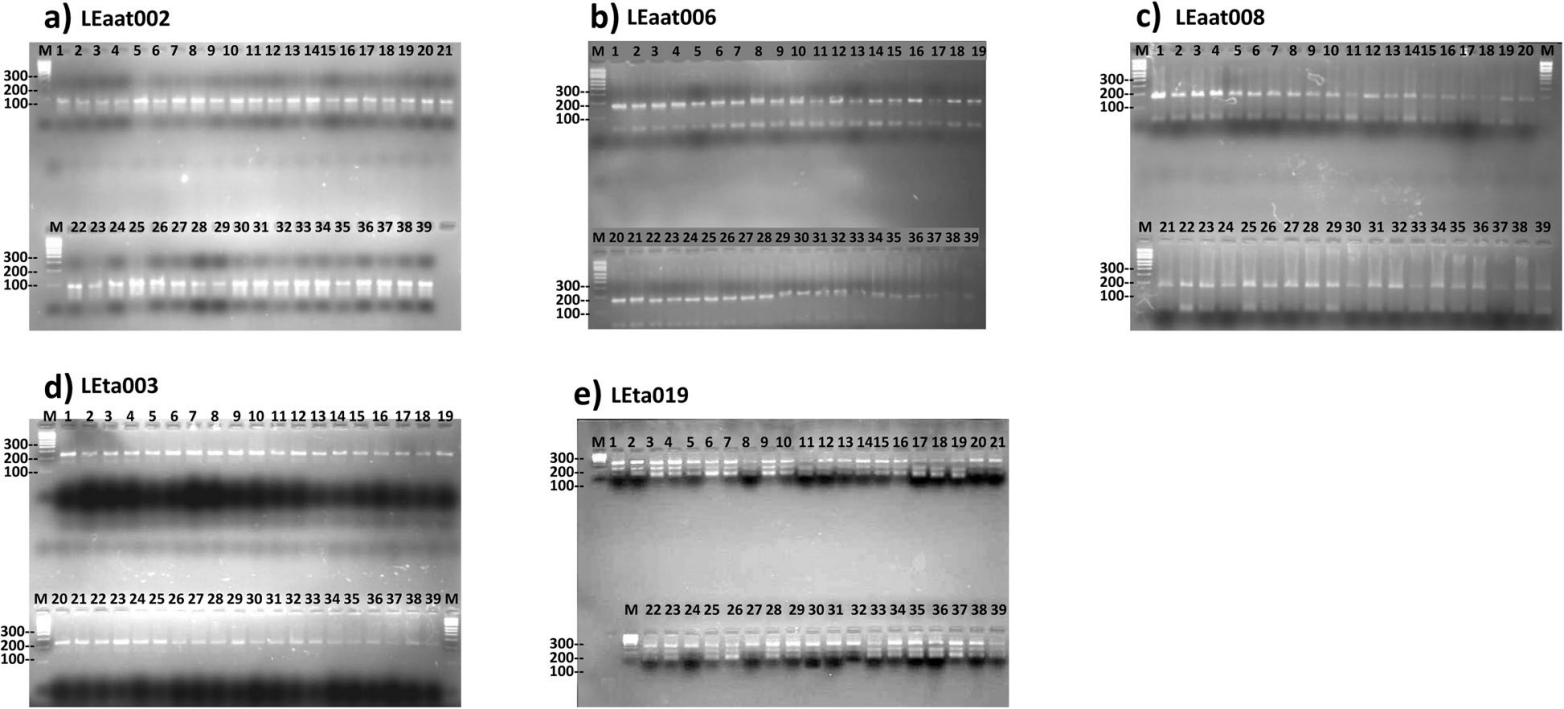
SSR pattern using primer LEaat002 (expected size is 106 bp), LEaat006 (expected size is 174 bp), LEaat008 (expected size is 178 bp), LEga003 (expected size is 241 bp), and LEta019 (Expected size is 301 bp) for 39 tomato landraces. The numbers from 1 to 39 denote tomato landrace number. M = molecular weight marker (100bp)

Thirty-nine tomato landraces collected from different regions were screened using seventeen SSR primers (Table 1). Five of the seventeen SSR primer pairs were able to produce the expected DNA fragments in their PCR products, while the remaining twelve primers were unable to amplify the expected PCR fragments. In this context, the genetic relationship among 39 tomato landraces was analyzed using these five primers. A total of nineteen loci (346 alleles) were produced where eighteen of these loci (95%) were polymorphic (Table 2). The banding patterns of SSR are shown in Fig. 3. The total number of loci produced with each primer ranged from 2 for Primer LEga 003 to 6 for Primer LEta019. The total percentage of polymorphism was 95% and the percentage of polymorphism ranged from 100% for primers LEaat002, LEaat006, LEaat008, LEta019 to 50% for primer (LEga003).

The genetic variation between tomato landraces was assessed using the Nie genetic distance and the UPGMA. The dissimilarity among thirty-nine tomato landraces ranged from 0.04 for tomato accessions number 25 (Jo969) and 26 (Jo981); 29 (Jo964) and 30 (Jo959) to 1.45 for tomato accessions number 3 (Jo960) and 39 (Jo980A); 23 (Jo978) and 39 (Jo980A).

**Fig. 4:**
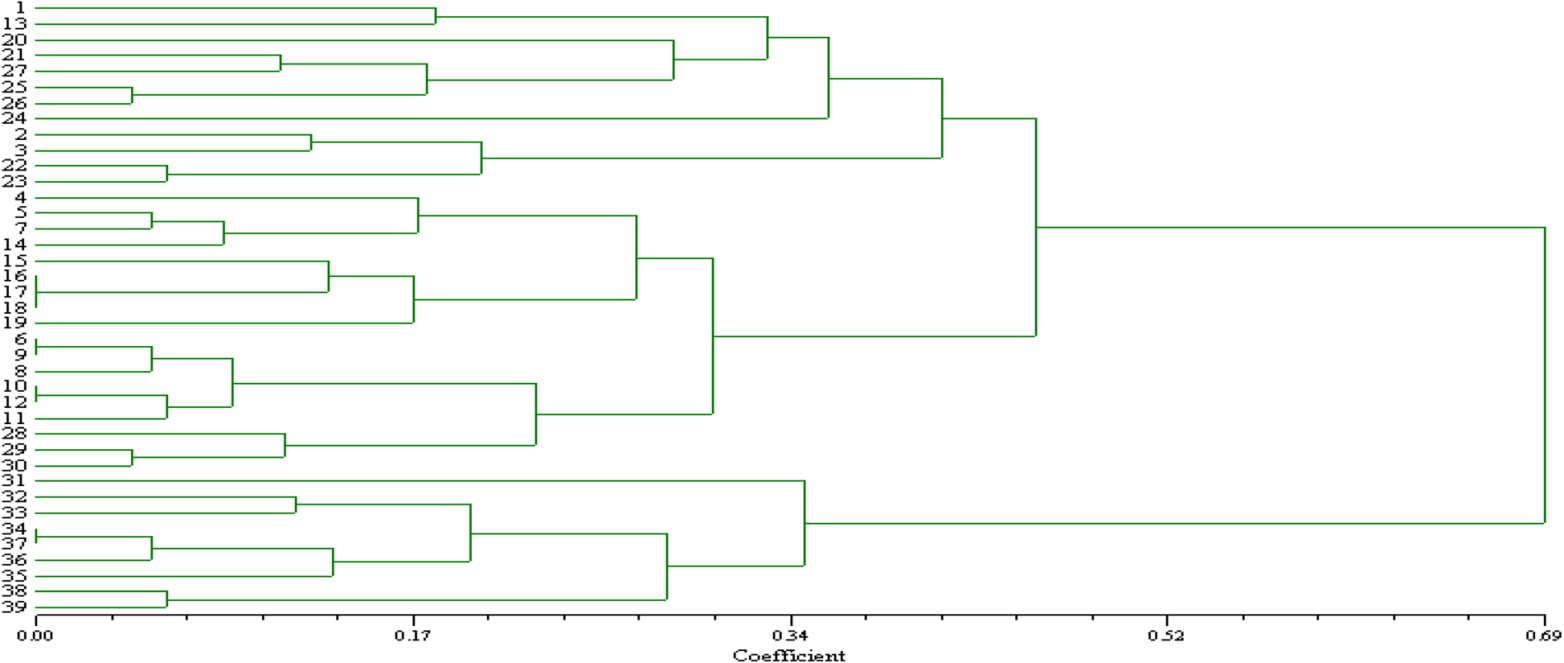
Dendrogram of 39 tomato landraces generated by UPGMA cluster analysis of the dissimilarity values based on Nei (1972) coefficient

UPGMA dendrogram of thirty-nine tomato landraces was constructed from the similarity value, which separated into two main clusters (Fig. 4). The first main cluster includes 30 landraces (tomato accession number 1 to accession number 30) with a dissimilarity value of (45 %) while the remaining tomato accession number (31-39) formed the second cluster with a dissimilarity value of (35%). According to Saal, Wricke [36], the average PIC was 0.998, ranging from 0.996 for primer LEaat006 to 0.998 for primers LEaat002, LEaat008, LEga003, and LEta019.

### Functional annotations of the gene-associated with SSRs in tomato

The five primer pairs (LEaat002, LEaat006, LEaat008, LEga003, and LEta019, Table 3) may be linked to four genes. We conducted annotations for these four genes using various databases, including Phytozome, NCBI, InterPro, and KEGG, to predict their potential functions. In context, these genes were linked to a variety of processes, indicating that these gene-associated SSRs could be related to several essential biological functions, such as the LEaat002 and LEaat008 SSR markers, which were linked to MEIS1 Transcription factors genes (*Solyc07g007120* and *Solyc07g007120.2*) (Table 4). Also, the LEaat006 SSR marker was associated with Trypsin and protease inhibitor (Kunitz_legume) gene (*Solyc03g020010*). Moreover, the Carbonic anhydrase gene (*Solyc09g010970*) was associated with LEga 003 SSR marker. While the LEta019 SSR marker was associated with *S. lycopersicum* microsatellite repeat DNA region Unknown Protein (AHRD V1) gene (Table 4).

**Table 3:** SSRs name, total number of alleles, number of loci, polymorphic loci, percentage of polymorphism, and polymorphism information content (PIC)

SSR name	Total no. of allele	No. of loci	Polymorphicloci	Polymorphism %	PIC
LEaat002	55	4	4	100	0.998
LEaat006	48	3	3	100	0.996
LEaat008	40	4	4	100	0.998
LEga003	70	2	1	50	0.998
LEta019	134	6	6	100	0.998
Total	346	19	18		
Mean	69.4	3.8	3.6	95%	0.998

**Table 4:** BLAST corresponding Solyc (*Solanum lycopersicum*) gene sequences and annotation for SSR marker sequences

SSR name	Solyc gene	Annotation result
LEaat002	*Solyc07g007120*	Transcription factor MEIS1 and related HOX domain protein HOMEOBOX PROTEIN KNOTTED-1-LIKE 3-RELATED; contains Interpro domain(s) IPR005539 (ELK domain), IPR001356 (Homeobox domain), IPR009057 (Homeodomain-like), IPR005541 (KNOX2), IPR005540 (KNOX1), IPR008422 (Homeobox KN domain)
LEaat006	*Solyc03g020010*	Trypsin and protease inhibitor (Kunitz_legume); contains Interpro domain(s) IPR002160 (Proteinase inhibitor I3, Kunitz legume) and IPR011065 (Kunitz inhibitor ST1-like)
LEaat008	*Solyc07g007120.2*	Transcription factor MEIS1 and related HOX domain protein HOMEOBOX PROTEIN KNOTTED-1-LIKE 3-RELATED; contains Interpro domain(s) IPR005539 (ELK domain), IPR001356 (Homeobox domain), IPR009057 (Homeodomain-like), IPR005541 (KNOX2), IPR005540 (KNOX1), IPR008422 (Homeobox KN domain)
LEga003	*Solyc09g010970*	Carbonic anhydrase (EC 4.2.1.1); contains Interpro domain(s) IPR001765 carbonic anhydrases
LEta019	*Solyc09g042380*	*L. esculentum* microsatellite repeat DNA region Unknown Protein (AHRD V1)

### Expression pattern of the targeted genes in tomato tissue

**Fig. 5:**
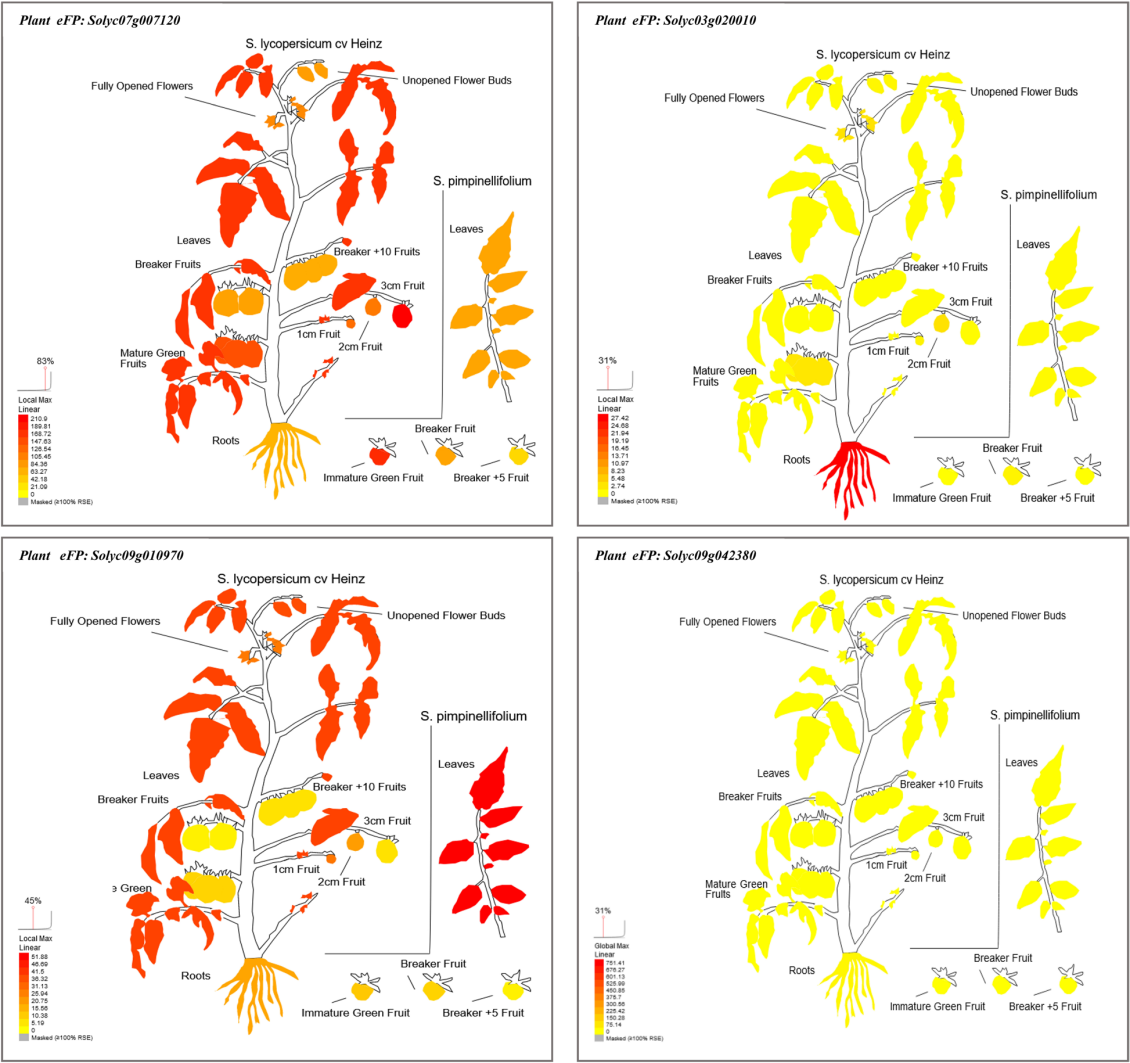
Visualization of the putative a “plant electronic fluorescent pictograph” browsers for exploring the putative tissue expression of *Solyc07g007120*, *Solyc03g020010*, *Solyc09g010970*, and *Solyc09g042380* genes, based on tomato gene expression and protein localization at different tissues and developmental stages. The bar represents the expression scale (the more intense the red color, the more gene expression) http://bar.utoronto.ca/eplant_tomato/

We analyzed gene expression profile maps of our target genes based on *S. lycopersicum* transcript expression database for further understanding the functions of our genes at different nineteen tissues (Fig. 5). Expression profiles were built using the tomato Electronic Fluorescent Pictograph Browsers (tomato eFP browsers (http://bar.utoronto.ca/eplant_tomato/). The arrow points to the expression scale (the more intense the red color, the more gene expression). It is clear from the Tomato Electronic Fluorescent Pictograph Expression Profiles Browsers that genes *Solyc07g007120* and *Solyc07g007120.2* which are related to the Transcription factors MEIS1 and have HOX domain protein homeobox protein knotted-1-like 3-related were highly expressed in all tomato tissues especially 3 cm fruit, pimpinellifolium immature green fruit and leaves (Fig. 5 and Table 4). While the highest expression levels for *Solyc03g020010* gene concerning Trypsin and protease inhibitor (Kunitz_legume) were observed at all of the tomato tissues such as root, 2 cm fruit, fully opened flower and mature green fruit (Fig. 5 and Table 4). Furthermore, the highest expression levels for *Solyc09g010970* gene which encodes carbonic anhydrase (EC 4.2.1.1) was recorded at pimpinellifolium leaf, leaves, unopened flower bud, 1 cm fruit, fully opened flower, 2 cm fruit and root (Fig. 5 and Table 4). On the contrary, *Solyc09g042380* gene which is related to *S. lycopersicum* microsatellite repeat DNA region unknown protein (AHRD V1) did not show any clear expression level at any tomato tissue.

### Subcellular localizations of the targeted genes in cell organelles

**Fig. 6:**
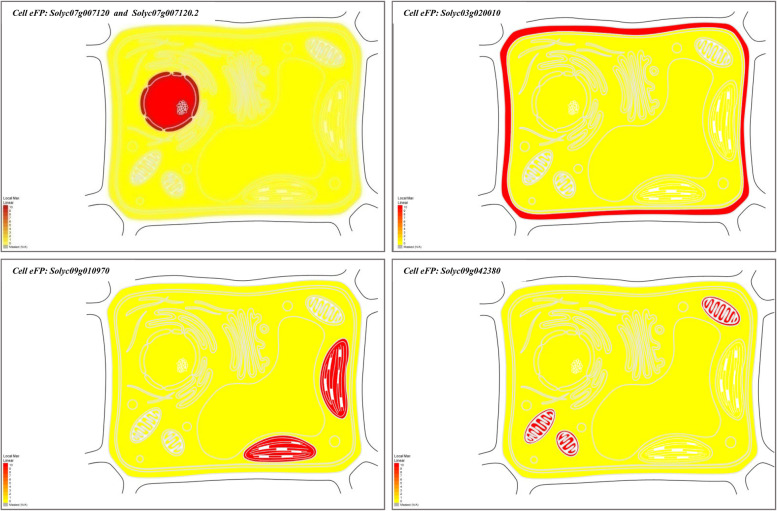
Putative subcellular localizations of our genes based on tomato protein localization at different cell organs. Cell sub-cellular localizations profile images were built using cell electronic fluorescent pictograph browsers (cell eFP browsers). The bar represents the expression scale (the more intense red color, the more gene expression), http://bar.utoronto.ca/eplant_tomato/

Here, we located the putative subcellular localizations of our target genes based on tomato protein localization at different cell organelles (such as cell plate, cytoskeleton, cytosol, endoplasmic reticulum, extracellular, golgi apparatus, mitochondrion, nucleus, peroxisome, plasma membrane, plastid, and vacuole). Putative cell subcellular localizations profile images were built using cell electronic fluorescent pictograph browsers (Cell eFP browsers (http://bar.utoronto.ca/eplant_tomato/)). It is clear from the Tomato Cell Electronic Fluorescent Pictograph subcellular localizations profiles that the *Solyc07g007120* and *Solyc07g007120.2* genes were highly expressed and presented in the nucleus (Fig. 6). Moreover, the *Solyc03g020010* and *Solyc09g010970* genes were highly expressed and presented in extracellular and plastid, respectively. While the *Solyc09g042380* did not detect any cell organelles (Fig. 6 and Table 4).

### The expression of the targeted genes in root cell types and tissues specific 

**Fig. 7:**
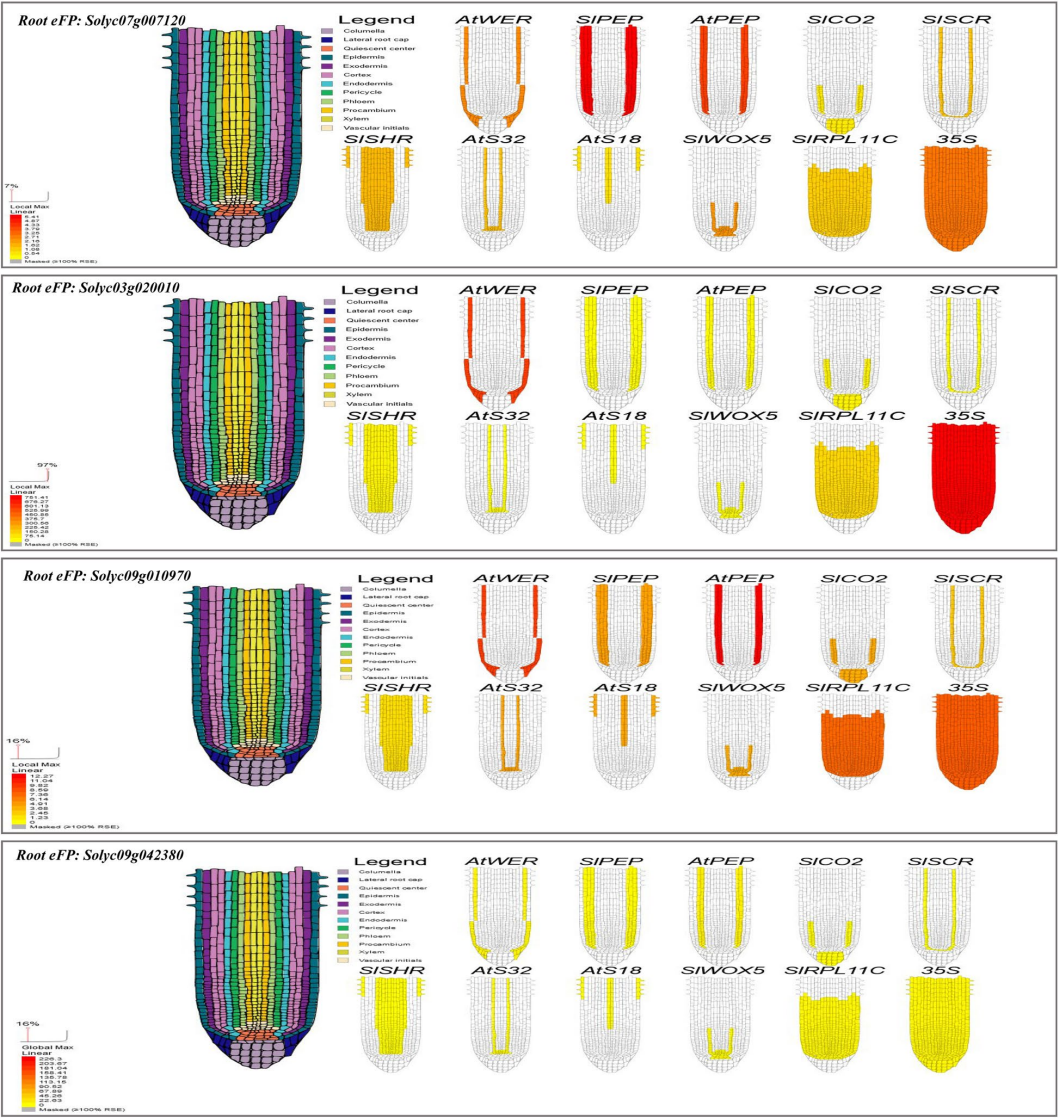
Visualization of the putative a “root electronic fluorescent pictograph” browsers for exploring the putative expression of *Solyc07g007120, Solyc03g020010, Solyc09g010970*, and *Solyc09g042380* genes at different root cell types- and tissues-specific under various promoter toolboxes, such as *AtWER, SIPEP, AtPEP, SICO2, SISCR*, *SISHR*, *AtS32*, *AtS18*, *SIWOX5*, *SIRPL11C*, and *35S* promoters. The bar represents the expression scale (the more intense the red color, the more gene expression) http://bar.utoronto.ca/eplant_tomato/

To predict the putative function of our candidate genes in root cell types- and tissues-specific (such as columella, lateral root cap, quiescent center, epidermis, exodermis, cortex, endodermis, pericycle, phloem, procambium, xylem, and vascular initials), we used root electronic fluorescent pictograph browsers (root FP browsers (http://bar.utoronto.ca/eplant_tomato/) to build the profile images under the effect of various promoters (Such as AtWER, SIPEP, AtPEP, SICO2, SISCR, SISHR, AtS32, AtS18, SIWOX5, SIRPL11C, and 35S). Interestingly, we observed the highest expression levels of *Solyc07g007120* and *Solyc07g007120*.2 genes in Exodermis and Cortex under the effect of SIPEPpro, followed by Cortex under AtPEPpro, all root cell types under 35Spro then Exodermis and Lateral root cap under AtWERpro (Fig. 7 and Table 4). Moreover, *Solyc03g020010* gene was highly expressed in all root cell types under 35Spro, then Exodermis and lateral root cap under AtWERpro. Also, highly expressed of *Solyc09g010970* gene was observed in the Cortex under AtPEPpro, followed by exodermis and lateral root cap under AtWERpro, then all root cell types under 35Spro. While the expression level of *Solyc09g042380* gene did not detect at any root cell types- and tissues-specific (Fig. 7 and Table 4).

## Discussion

Salinity stress has a negative influence on the tomato plant at different growth and developmental stages and phases e.g. seed germination, growth, biomass allocation, and fruit yield [7, 39, 40]. Our study revealed that there are significant differences among tomato landraces during seedling growth at 4 and 6 dS m^−1^ salinity levels, indicating that there is high genetic variation among them [41]. A highly significant reduction in shoot and root growth was detected among tomato landraces under salt stress. These findings agreed with those of Hajer et al. [42], who attributed the decrease in shoot and root growth in response to salt stress to lower osmotic pressure, which reduces water potential in root and shoot, or to oxidative stress.

In this study, regardless of landraces, increase Na^+^ and Cl^-^ content in tomato shoot is associated with a decrease in shoot fresh weight and dry weight [41]. Similarly, Alian et al. [43] found both elements accumulate with increasing salinity, and their accumulation is associated with a reduction in shoot fresh and dry weights. A high level of Na^+^ and Cl^-^ can cause ion-toxicity and nutrient imbalance, particularly concerning K^+^, Ca^2+^, and Mg^2+^, by interfering with their accumulation to the shoots [9, 44] while other authors attributed this to the reduction in water uptake [45]. In this study, nitrogen varies among the 14 landraces at 6 dS m^−1^, accession 29 (Jo964) has the highest N% while accession 38 (Jo955) has the lowest N%. According to Kafkafi et al. [46], salt-tolerant tomato cultivars have higher nitrogen levels than sensitive cultivars. A similar result was found with accession 29 (Jo964) where has an increase in nitrogen content as compared with the control. Also, K^+^ is decreased with salinity except in accession 24 (Jo970) where K^+^ content increased as compared with the control. Accession 38(Jo955) has the lowest value (3.64%). Tavakkoli et al. [47] found that the reduction of K^+^ content might be related to the competition between the ionic Na^+^ and K^+^ on the root absorptive sites. This decrease in K^+^ content in plant leaves leads to a decrease in growth by reducing plants’ ability to adjust to osmotic pressure [47, 48]. Calcium plays an important role in the regulation of membrane permeability and the strengthening of cell walls [48]. Calcium (Ca^2+^) content decreased with salinity stress and the difference in Ca^2+^ content among landraces may be due to the difference in Ca^2+^ displacement by Na^+^ from the cell membrane.

Detecting high similarity in proline content among accessions 36 (Jo987), 37 (Jo957), 39 (Jo980A), 31 (Jo976), 34 (Jo985), and 21 (Jo974A), 24 (Jo970) were not surprising, since the highest similarity was found at DNA levels were detected in this study. This finding is confirmed by cluster analysis where accession is grouped in two main clusters. In tomato plants, Fariba, Ali [49] reported that the increment in proline content in response to salt stress might be attributed to the expression of genes encoding key enzymes of proline synthesis Pyrroline-5-carboxylate (P5C) and low activity of the oxidizing enzyme (Proline dehydrogenase).

### Genetic variation among tomato landraces

The number of polymorphic loci for the primers used in this study was ranged from one locus for primer LEga003 to three loci for primer LEta019. Genetic variation as polymorphism percentage was high among thirty-nine tomato landraces which are nearly similar to the results reported before [50]. The genetic distance between the studied tomato landraces was constructed and significant differences in the dissimilarity values were also detected which may be attributed to differences in their shape and their origin. For instance, landrace 39 (Jo 980A) was rounded and originated from Afra (Southern Jordan) while landrace 3 (Jo 960) was slightly flattened and originated from Shatanah (Northern Jordan). Tomato landraces clustering based on the genetic distance showed group includes thirty accessions that have small to large fruit sizes whereas the second cluster includes nine accessions with a very small to intermediate fruit size. A wide variation of PIC was found for primer LEaat006 and primers LEaat002, LEaat008, LEga003, LEta019, respectively. Previous studies by He et al. [31] and Tam et al. [50] found a wide range of PIC in tomato worldwide cultivars collections. In general, the wide range of genetic distance indicated high DNA polymorphism occurred among tomato landraces which indicated high genetic variation among landraces and the present study confirms the usefulness of SSR markers as a powerful tool or revealed genetic variation among tomato landraces.

### Putative tissue expression pattern, subcellular localization, root cell types, and tissues specific of the targeted genes-associated SSR markers

The functional annotations analysis of the gene-associated with SSRs in tomato detected four genes which we used for gene expression at different tissues, subcellular localizations, root cell types- and tissues-specific in *S. lycopersicum* and *S. pennellii*. Furthermore, a putative expression and recognized synthesis sites of these genes provide a good way for understanding the epistatic relationship between our genes synthesis site and putative functions. For example, LEaat002 and LEaat008 SSR markers were associated with MEIS1 Transcription factors genes (*Solyc07g007120* and *Solyc07g007120*.2) and this Transcription factors gene has HOX domain protein HOMEOBOX PROTEIN KNOTTED-1-LIKE 3-RELATED that contains several Interpro domains such as IPR005539 (ELK domain), IPR001356 (Homeobox domain), IPR009057 (Homeodomain-like), IPR005541 (KNOX2), IPR005540 (KNOX1), and IPR008422 (Homeobox KN domain). All of the previous Interpro domains are known to be key regulators for plant development and can be binding with DNA through a helix-turn-helix (HTH) structure [51–53]. In context, these domains have roles in the development of the shoot apical meristem (SAM) [54–56], whereas gain-of-function mutants of Knox domain in tomato (*S. lycopersicum*) produce different phenotypic traits with modified leaf morphological characteristics [57, 58]. Moreover, we found these two genes were highly expression at different plant tissue, for instance, exodermis, cortex, lateral root cap, and all root cell types under AtPEPpro, 35Spro SIPEPpro, and AtWERpro suggest that these genes are potentially involved in different root cell types such as the elongation zone and meristematic zone figure [59]. A previous study conducted by Kerstetter et al. [53], found some of these domains have meristem-specific mRNA expression patterns, while others have a more prevalent expression pattern. Sakamoto et al. [60] reported that these domains can suppress target gene expression by acting as a nuclear localization signal. Furthermore, the LEaat006 SSR marker was associated with Trypsin and protease inhibitor (Kunitz_legume) gene (*Solyc03g020010*) and this gene contains two Interpro domains such as IPR002160 (Proteinase inhibitor I3, Kunitz legume) and IPR011065 (Kunitz inhibitor ST1-like). These domains belong to the MEROPS inhibitor family that can exhibit proteinase inhibitory activity against serine proteinases and are responsible for binding to sensitive cells found on the C-terminal of the heavy chain [61]. On the other hand, based on our results, this gene is highly expressed in different tissues including all root cell types under 35Spro and AtWERpro which could be involved in root developmental mechanisms such as the elongation and meristematic zones figure. This finding agreed with Shan et al. [62] who indicated the link between upregulation of the WRSI5 gene, which includes the Proteinase inhibitor domain, and salt tolerance in wheat. In *Arabidopsis thaliana*, overexpression of the WRSI5 gene can also improve seedling growth on a medium containing 150 mM NaCl by increasing K^+^ ion selectivity over Na^+^ ion through the mature root tips. The results of the current study are in agreement with Srinivasan et al. [63] who reported the overexpression of the trypsin protease inhibitor gene under the 35S promoter can exhibit tolerance to salinity in the form of NaCl, variable pH, and sorbitol in transgenic tobacco through exhibiting higher K^+^ ions and an optimum Na^+^/K^+^ ratio.

Further, LEga 003 SSR marker was associated with Carbonic anhydrase (CAs; EC 4.2.1.1) gene (*Solyc09g010970*) which contains Interpro domain IPR001765 carbonic anhydrases. This domain can catalyze the reversible hydration of carbon dioxide and have an essential role in inorganic carbon fixation through photosynthetic in plant chloroplast [64, 65]. Here, we found that this gene was expressed highly in tomato root tissues, for example, cortex, lateral root cap, and other root cell types suggesting its importance as root-specific tissues and involved in root elongation and meristematic development. These results are in line with DiMario et al. [66] that found CAs are expressed in numerous plant tissues and different cellular locations (such as chloroplast, cytosol, and mitochondria). Moreover, carbonic anhydrases (CAs) are a class of Zn-containing enzymes that catalyze the reversible hydration of carbon dioxide while producing protons and bicarbonate that are involved in several metabolic processes in different plant species. Furthermore, the CAs play an essential role in photosynthesis in both C3 and C4 plants, signaling, activation of several protective response genes, and protection of crop plants in response to several stress conditions including high illumination, low and high concentration of carbon dioxide, drought, and salinity [66–68].

## Conclusions

In conclusion, the tomato has been cataloged as moderately sensitive to salinity at all stages of plant development, including seed germination, vegetative growth, reproduction, and finally yield losses. We demonstrated the sígnifcant influence of salinity at levels (4 and 6 dS m^-1^) on seedling-related traits in all tested tomato landraces. Moreover, a wide range of genetic distances indicated high DNA polymorphism occurred among tomato landraces, elucidating the high genetic variation among landraces. The present study confirms the usefulness of SSR markers as a powerful tool to reveal the genetic variation among tomato landraces. On the other hand, we had predicted the putative tissue expression pattern, subcellular localization, root cell types- and tissues-specific of our target genes-associated SSR loci. Furthermore, through the expression pattern profile for our candidate genes, can answer biological questions related to the function of our candidate genes and their associated SSR loci in specific tissues, cell organs, and root cell type-specific and their adaptive potential under salt stress. Further molecular genetic and breeding application based upon the outputs is important for tomato performance and yield improvement.

## Supplementary Information

**Additional file 1: Table S1.** Relative Reduction (R. R. %) in shoot and root fresh and dry weights, shoot and root growth rates and the relative Increase (R. I. %) of proline content in response to 4 and 6 dS m^1^ֿ salinity level. **Table S2.** Analysis of Variance (ANOVA) for relative reduction of shoot fresh weight of seedling tomato landraces (4 dS mֿ^1^) salinity level. **Table S3.** Analysis of Variance (ANOVA) for relative reduction of shoot dry weight of seedling tomato landraces (4 dS mֿ^1^) salinity level. **Table S4.** Analysis of Variance (ANOVA) for relative reduction of root fresh weight of seedling tomato landraces (4 dS mֿ^1^) salinity level. **Table S5.** Analysis of Variance (ANOVA) for relative reduction of root growth rate of seedling tomato landraces (4 dS mֿ^1^) salinity level. **Table S6.** Analysis of Variance (ANOVA) for relative reduction of shoot fresh weight of seedling tomato landraces (6 dS mֿ^1^) salinity level. **Table S7.** Analysis of Variance (ANOVA) for relative reduction of shoot dry weight of seedling tomato landraces (6 dS mֿ^1^) salinity level. **Table S8.** Analysis of Variance (ANOVA) for relative reduction of root dry weight of seedling tomato landraces (4 dS mֿ^1^) salinity level. **Table S9.** Analysis of Variance (ANOVA) for relative reduction of root dry weight of seedling tomato landraces (6 dS mֿ^1^) salinity level. **Table S10.** Analysis of Variance (ANOVA) for relative reduction of shoot growth rate of seedling tomato landraces ( 6 dS mֿ^1^) salinity level. **Table S11.** Analysis of Variance (ANOVA) for relative reduction of root fresh weight of seedling tomato landraces ( 6 dS mֿ^1^) salinity level. **Table S12.** Analysis of Variance (ANOVA) for relative reduction of root growth rate of seedling tomato landraces( 6 dS mֿ^1^) salinity level. **Table S13.** Analysis of Variance (ANOVA) for relative increase of leave proline of seedling tomato landraces at salinity level( 4 dS mֿ^1^) salinity level. **Table S14.** Analysis of Variance (ANOVA) for relative increase of leave proline of seedling tomato landraces at salinity level (6 dS mֿ^1^) salinity level. **Table S15.** Minerals content (% of dry weight) of tomato shoot and roots of 39 accessions grown at control and 4 dSm^-^1 salinity level. **Table S16.** Minerals content (% of dry weight) of tomato shoots and roots of 14 accessions grown at 6 dS m^-^1 salinity level. **Table S17.** T-test for mineral content in shoot of 34 tomato accessions compared (1) control and (2) 4 dS mֿ^1^ salinity level. **Table S18.** T-test for mineral content in root of 34 tomato accessions compared (1) control and (2) salinity level 4 dS mֿ^1^.**Table S19.** T-test for mineral content in shoot of 14 tomato accessions compared (1) control (2) 4dS mֿ^1^ and (3) 6 dS mֿ^1^ salinity level. **Table S20.** T-test for mineral content in root of 14 tomato accessions compared (1) control and (2) level 4 dS mֿ^1^ and (3) level 6 dS mֿ^1^.

**Additional file 2: Figure S1.** Unweighted Pair Group Method with Arithmetic average (UPGMA) dendrogram of the genetic dissimilarity of the tomato accessions based on Euclidean distance coefficient using proline content at 4 dS mֿ^1^ salinity level. 1. Jo111A, 2. Jo 111B, 3. Jo 960, 5. Jo 952, 6. Jo 956, 8. Jo 972, 9. Jo 973 11. Jo 967B, 12. 9 Jo 71A, 13. Jo 971B, 14. Jo 961, 15. Jo 979, 17. Jo 989, 18. Jo 968, 19. Jo 958, 20. Jo 974B, 21. Jo 974A, 22. Jo 994A, 23. Jo 978, 24. Jo 970, 25. Jo 969, 26. Jo 981, 27. Jo 991A, 28. Jo 991B, 29. Jo 964, 30. Jo 959, 31. Jo 976, 32. Jo 975, 33. Jo 963, 34. Jo 985, 36. Jo 987, 37. Jo 957, 38. Jo 955, 39. Jo 980A. **Figure S2.** UPGMA dendrogram of the genetic dissimilarity of the tomato accessions based on Euclidean distance coefficient using proline content at 6 dS mֿ^1^ salinity level. 21. Jo 974A, 23. Jo 978, 24. Jo 970, 25, Jo 969, 29. Jo 964, 30. Jo 959, 31. Jo 976, 32. Jo 975, 33. Jo 963, 34. Jo 985, 36. Jo 987, 38. Jo 955, 39. Jo 980A, 37. Jo 957. **Figure S3.** UPGMA dendrogram of the genetic dissimilarity of the tomato accessions based on Euclidean distance coefficient using shoot mineral content at 4 dS mֿ^1^ salinity level. Jo111A, 2. Jo 111B, 3. Jo 960, 5. Jo 952, 6. Jo 956, 8. Jo 972, 9. Jo 973 11. Jo 967B, 12. 9 Jo 71A, 13. Jo 971B, 14. Jo 961, 15. Jo 979, 17. Jo 989, 18. Jo 968, 19. Jo 958, 20. Jo 974B, 21. Jo 974A, 22. Jo 994A, 23. Jo 978, 24. Jo 970, 25. Jo 969, 26. Jo 981, 27. Jo 991A, 28. Jo 991B, 29. Jo 964, 30. Jo 959, 31. Jo 976, 32. Jo 975, 33. Jo 963, 34. Jo 985, 36. Jo 987, 37. Jo 957, 38. Jo 955, 39. Jo 980A. **Figure S4.** UPGMA dendrogram of the genetic dissimilarity of the tomato accessions based on Euclidean distance coefficient using shoot mineral content at 6 dS mֿ^1^ salinity level. 21. Jo 974A, 23. Jo 978, 24. Jo 970, 25, Jo 969, 29. Jo 964, 30. Jo 959, 31. Jo 976, 32. Jo 975, 33. Jo 963, 34. Jo 985, 36. Jo 987, 38. Jo 955, 39. Jo 980A, 37. Jo 957. **Figure S5.** UPGMA dendrogram of the genetic dissimilarity of the tomato accessions based on Euclidean distance coefficient using root mineral content at 4 dS mֿ^1^ salinity level. Jo111A, 2. Jo 111B, 3. Jo 960, 5. Jo 952, 6. Jo 956, 8. Jo 972, 9. Jo 973 11. Jo 967B, 12. 9 Jo 71A, 13. Jo 971B, 14. Jo 961, 15. Jo 979, 17. Jo 989, 18. Jo 968, 19. Jo 958, 20. Jo 974B, 21. Jo 974A, 22. Jo 994A, 23. Jo 978, 24. Jo 970, 25. Jo 969, 26. Jo 981, 27. Jo 991A, 28. Jo 991B, 29. Jo 964, 30. Jo 959, 31. Jo 976, 32. Jo 975, 33. Jo 963, 34. Jo 985, 36. Jo 987, 37. Jo 957, 38. Jo 955, 39. Jo 980A. **Figure S6.** UPGMA dendrogram of the genetic dissimilarity of the tomato accessions based on Euclidean distance coefficient using root mineral content at 6 dS mֿ^1^ salinity level. 21. Jo 974A, 23. Jo 978, 24. Jo 970, 25, Jo 969, 29. Jo 964, 30. Jo 959, 31. Jo 976, 32. Jo 975, 33. Jo 963, 34. Jo 985, 36. Jo 987, 38. Jo 955, 39. Jo 980A, 37. Jo 957.

## Data Availability

The datasets used and/or analysed during the current study are available from the corresponding author on reasonable request.

## Abbreviations

RAPD: Random amplified polymorphic DNA
AFLP: Amplified fragment length polymorphism
SSR: Simple sequence repeats
PIC: Polymorphism information content
PCR: Polymerase chain reaction
UPGMA: Unweighted pair group method with arithmetic average
LSD: Least significant difference
N: Nitrogen
P: Phosphorus
K: Potassium
Ca: Calcium
Mg: Magnesium
Na: Sodium
Cl: Chloride
