# Supplementary material for: Exploring genetic variation among Jordanian Solanum lycopersicon L. landraces and their performance under salt stress using SSR markers
Source: J Genet Eng Biotechnol. 2022 Mar 11;20:45. doi: 10.1186/s43141-022-00327-2 (PMC8917245; doi:10.1186/s43141-022-00327-2)
Supplement: Supplementary file 2 — Additional file 2: Figure S1. Unweighted Pair Group Method with Arithmetic average (UPGMA) dendrogram of the genetic dissimilarity of the tomato accessions based on Euclidean distance coefficient using proline content at 4 dS mֿ1 salinity level. 1. Jo111A, 2. Jo 111B, 3. Jo 960, 5. Jo 952, 6. Jo 956, 8. Jo 972, 9. Jo 973 11. Jo 967B, 12. 9 Jo 71A, 13. Jo 971B, 14. Jo 961, 15. Jo 979, 17. Jo 989, 18. Jo 968, 19. Jo 958, 20. Jo 974B, 21. Jo 974A, 22. Jo 994A, 23. Jo 978, 24. Jo 970, 25. Jo 969, 26. Jo 981, 27. Jo 991A, 28. Jo 991B, 29. Jo 964, 30. Jo 959, 31. Jo 976, 32. Jo 975, 33. Jo 963, 34. Jo 985, 36. Jo 987, 37. Jo 957, 38. Jo 955, 39. Jo 980A. Figure S2. UPGMA dendrogram of the genetic dissimilarity of the tomato accessions based on Euclidean distance coefficient using proline content at 6 dS mֿ1 salinity level. 21. Jo 974A, 23. Jo 978, 24. Jo 970, 25, Jo 969, 29. Jo 964, 30. Jo 959, 31. Jo 976, 32. Jo 975, 33. Jo 963, 34. Jo 985, 36. Jo 987, 38. Jo 955, 39. Jo 980A, 37. Jo 957. Figure S3. UPGMA dendrogram of the genetic dissimilarity of the tomato accessions based on Euclidean distance coefficient using shoot mineral content at 4 dS mֿ1 salinity level. Jo111A, 2. Jo 111B, 3. Jo 960, 5. Jo 952, 6. Jo 956, 8. Jo 972, 9. Jo 973 11. Jo 967B, 12. 9 Jo 71A, 13. Jo 971B, 14. Jo 961, 15. Jo 979, 17. Jo 989, 18. Jo 968, 19. Jo 958, 20. Jo 974B, 21. Jo 974A, 22. Jo 994A, 23. Jo 978, 24. Jo 970, 25. Jo 969, 26. Jo 981, 27. Jo 991A, 28. Jo 991B, 29. Jo 964, 30. Jo 959, 31. Jo 976, 32. Jo 975, 33. Jo 963, 34. Jo 985, 36. Jo 987, 37. Jo 957, 38. Jo 955, 39. Jo 980A. Figure S4. UPGMA dendrogram of the genetic dissimilarity of the tomato accessions based on Euclidean distance coefficient using shoot mineral content at 6 dS mֿ1 salinity level. 21. Jo 974A, 23. Jo 978, 24. Jo 970, 25, Jo 969, 29. Jo 964, 30. Jo 959, 31. Jo 976, 32. Jo 975, 33. Jo 963, 34. Jo 985, 36. Jo 987, 38. Jo 955, 39. Jo 980A, 37. Jo 957. Figure S5. UPGMA dendrogram of the genetic dissimilarity of the tom [file 43141_2022_327_MOESM2_ESM.pptx]

## Slide 1
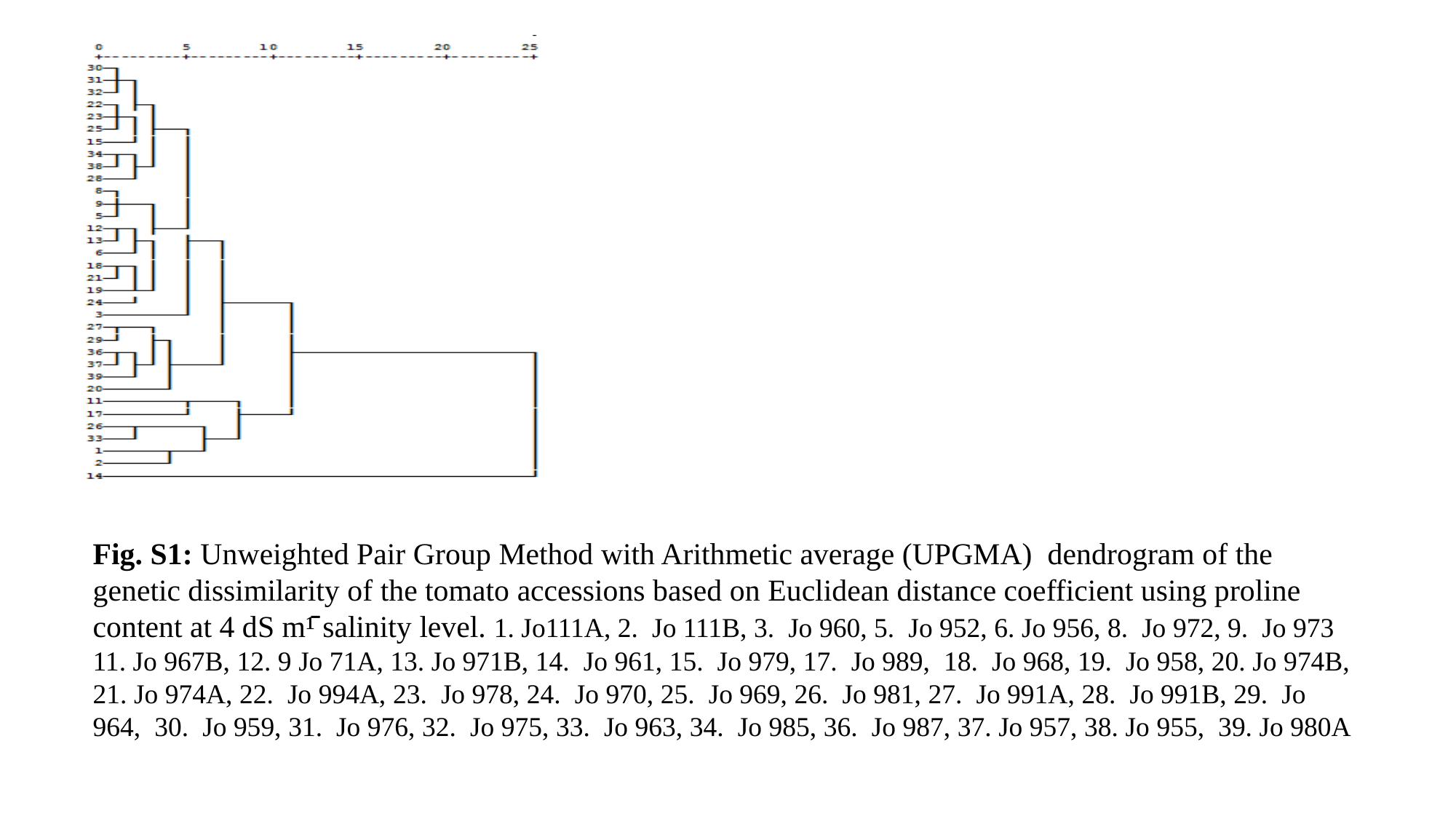

Fig. S1: Unweighted Pair Group Method with Arithmetic average (UPGMA) dendrogram of the genetic dissimilarity of the tomato accessions based on Euclidean distance coefficient using proline content at 4 dS mֿ¹ salinity level. 1. Jo111A, 2. Jo 111B, 3. Jo 960, 5. Jo 952, 6. Jo 956, 8. Jo 972, 9. Jo 973 11. Jo 967B, 12. 9 Jo 71A, 13. Jo 971B, 14. Jo 961, 15. Jo 979, 17. Jo 989, 18. Jo 968, 19. Jo 958, 20. Jo 974B, 21. Jo 974A, 22. Jo 994A, 23. Jo 978, 24. Jo 970, 25. Jo 969, 26. Jo 981, 27. Jo 991A, 28. Jo 991B, 29. Jo 964, 30. Jo 959, 31. Jo 976, 32. Jo 975, 33. Jo 963, 34. Jo 985, 36. Jo 987, 37. Jo 957, 38. Jo 955, 39. Jo 980A

## Slide 2
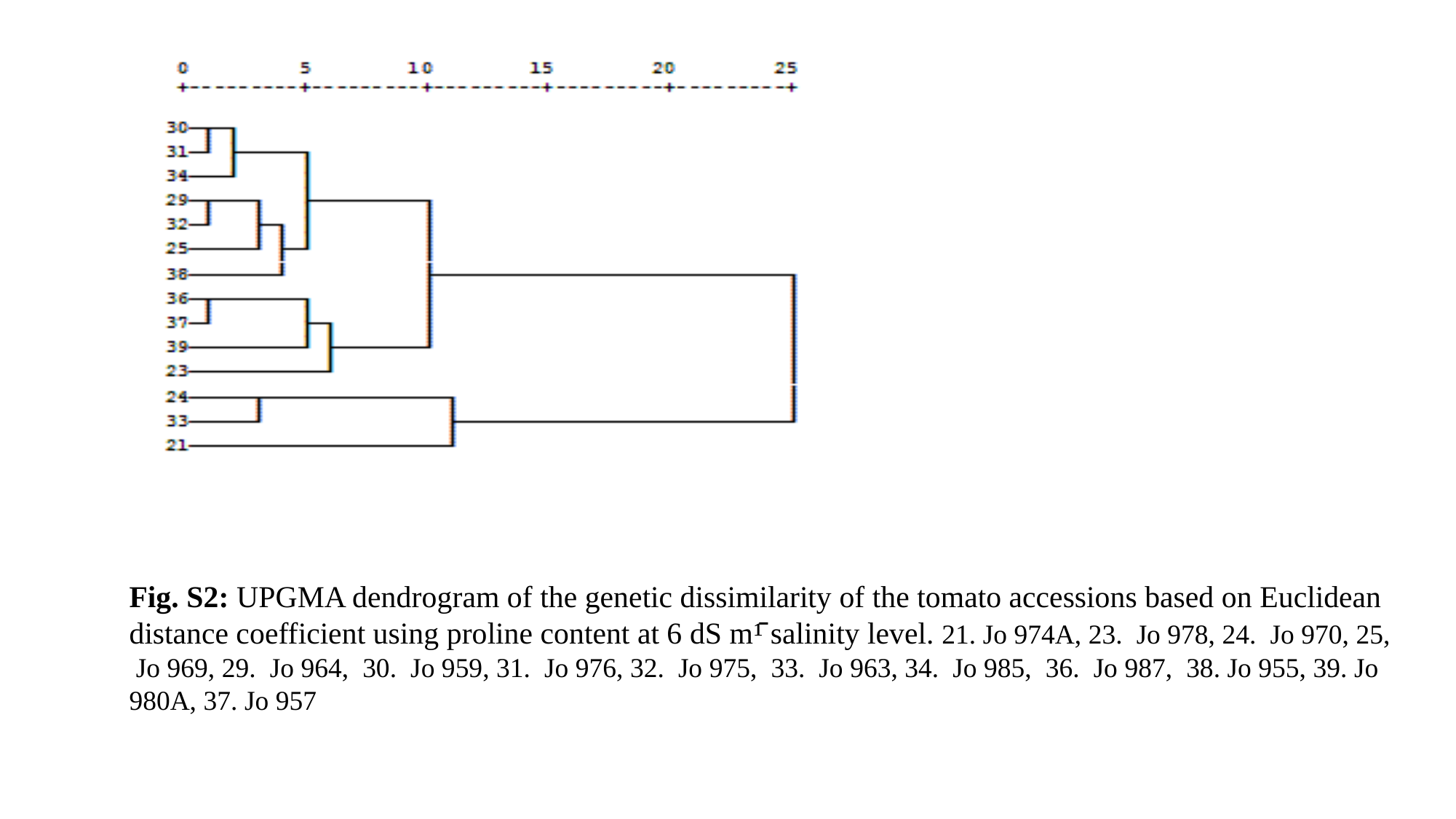

Fig. S2: UPGMA dendrogram of the genetic dissimilarity of the tomato accessions based on Euclidean distance coefficient using proline content at 6 dS mֿ¹ salinity level. 21. Jo 974A, 23. Jo 978, 24. Jo 970, 25, Jo 969, 29. Jo 964, 30. Jo 959, 31. Jo 976, 32. Jo 975, 33. Jo 963, 34. Jo 985, 36. Jo 987, 38. Jo 955, 39. Jo 980A, 37. Jo 957

## Slide 3
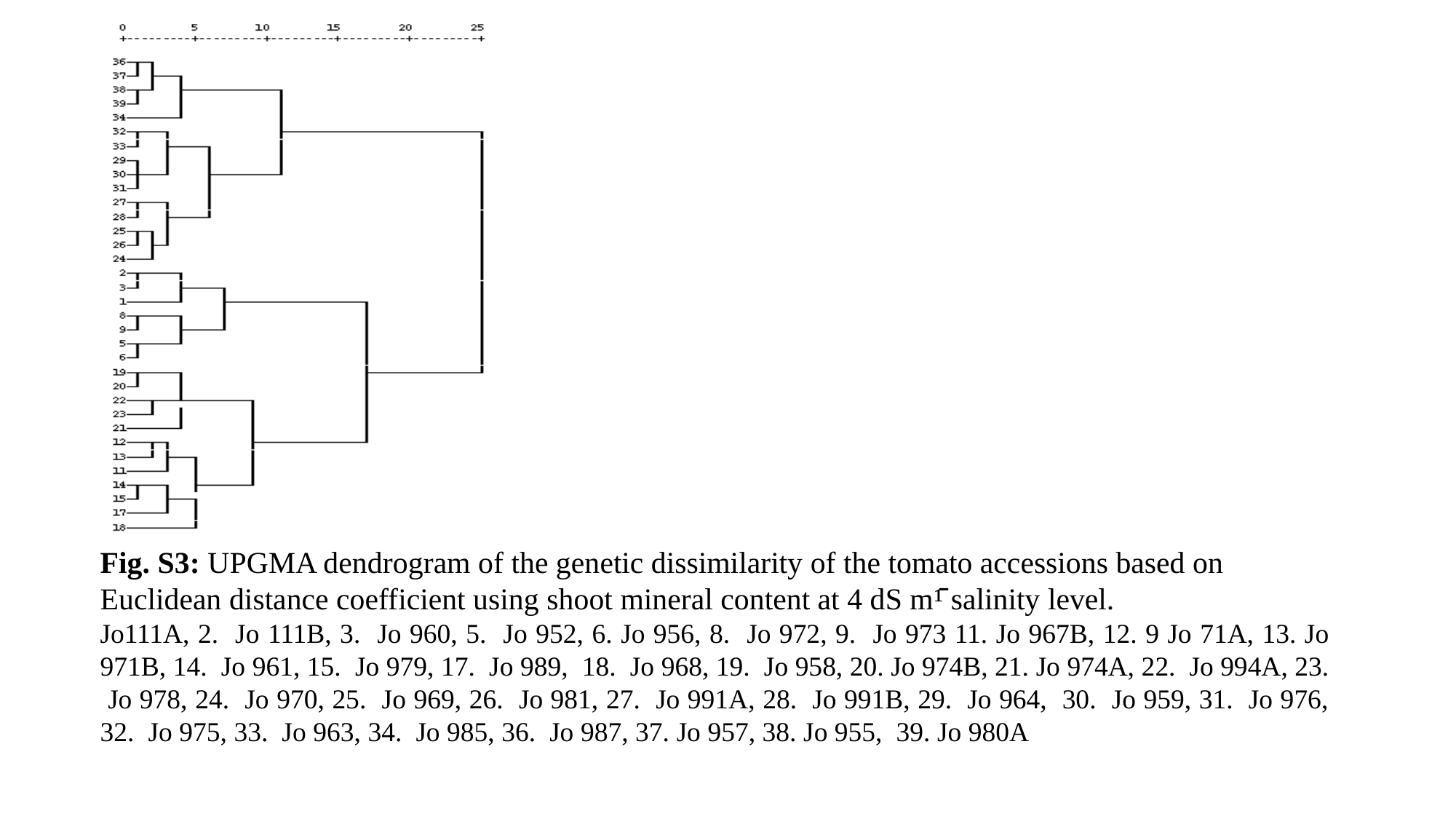

Fig. S3: UPGMA dendrogram of the genetic dissimilarity of the tomato accessions based on Euclidean distance coefficient using shoot mineral content at 4 dS mֿ¹ salinity level.
Jo111A, 2. Jo 111B, 3. Jo 960, 5. Jo 952, 6. Jo 956, 8. Jo 972, 9. Jo 973 11. Jo 967B, 12. 9 Jo 71A, 13. Jo 971B, 14. Jo 961, 15. Jo 979, 17. Jo 989, 18. Jo 968, 19. Jo 958, 20. Jo 974B, 21. Jo 974A, 22. Jo 994A, 23. Jo 978, 24. Jo 970, 25. Jo 969, 26. Jo 981, 27. Jo 991A, 28. Jo 991B, 29. Jo 964, 30. Jo 959, 31. Jo 976, 32. Jo 975, 33. Jo 963, 34. Jo 985, 36. Jo 987, 37. Jo 957, 38. Jo 955, 39. Jo 980A

## Slide 4
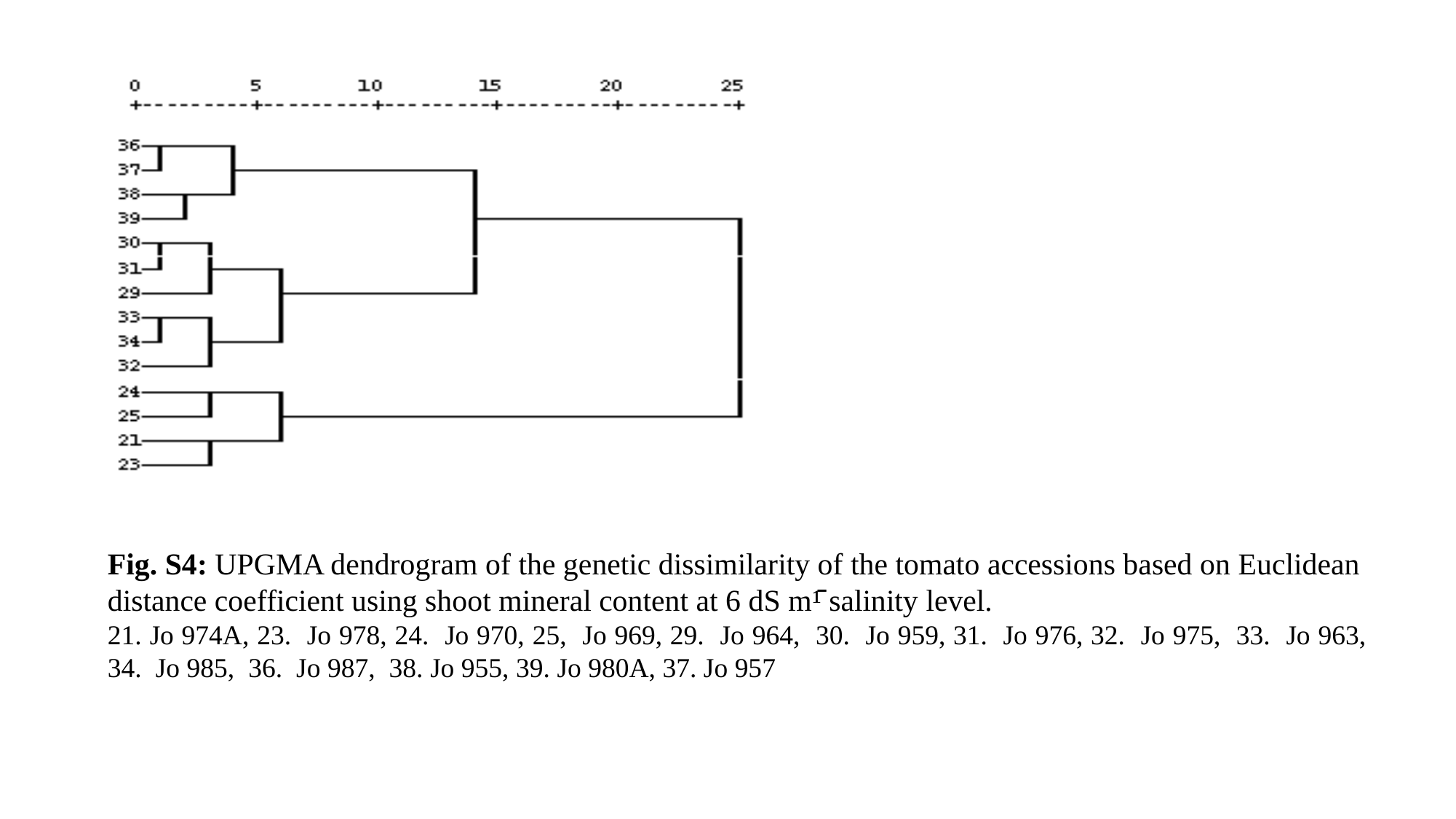

Fig. S4: UPGMA dendrogram of the genetic dissimilarity of the tomato accessions based on Euclidean distance coefficient using shoot mineral content at 6 dS mֿ¹ salinity level.
21. Jo 974A, 23. Jo 978, 24. Jo 970, 25, Jo 969, 29. Jo 964, 30. Jo 959, 31. Jo 976, 32. Jo 975, 33. Jo 963, 34. Jo 985, 36. Jo 987, 38. Jo 955, 39. Jo 980A, 37. Jo 957

## Slide 5
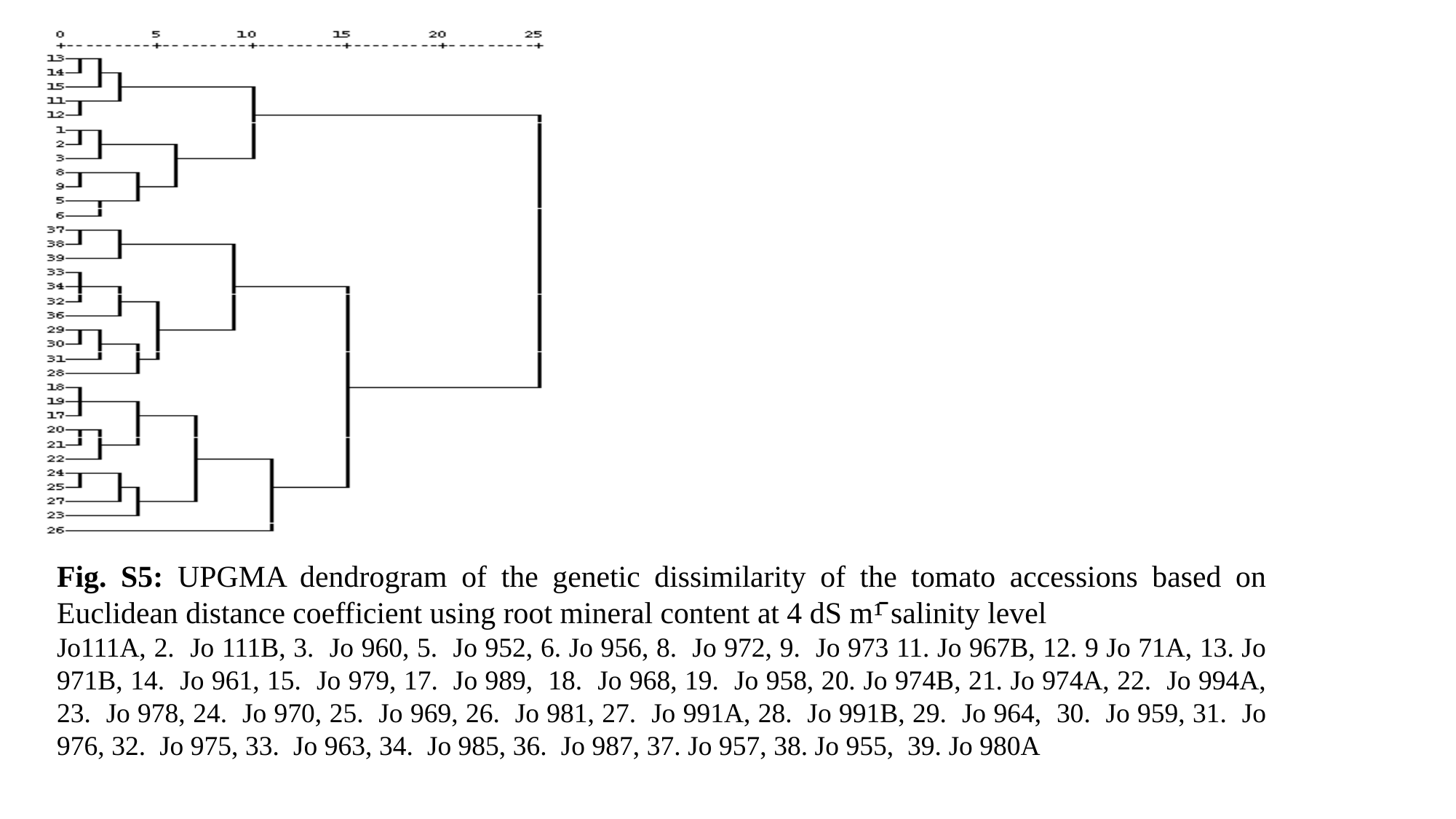

Fig. S5: UPGMA dendrogram of the genetic dissimilarity of the tomato accessions based on Euclidean distance coefficient using root mineral content at 4 dS mֿ¹ salinity level
Jo111A, 2. Jo 111B, 3. Jo 960, 5. Jo 952, 6. Jo 956, 8. Jo 972, 9. Jo 973 11. Jo 967B, 12. 9 Jo 71A, 13. Jo 971B, 14. Jo 961, 15. Jo 979, 17. Jo 989, 18. Jo 968, 19. Jo 958, 20. Jo 974B, 21. Jo 974A, 22. Jo 994A, 23. Jo 978, 24. Jo 970, 25. Jo 969, 26. Jo 981, 27. Jo 991A, 28. Jo 991B, 29. Jo 964, 30. Jo 959, 31. Jo 976, 32. Jo 975, 33. Jo 963, 34. Jo 985, 36. Jo 987, 37. Jo 957, 38. Jo 955, 39. Jo 980A

## Slide 6
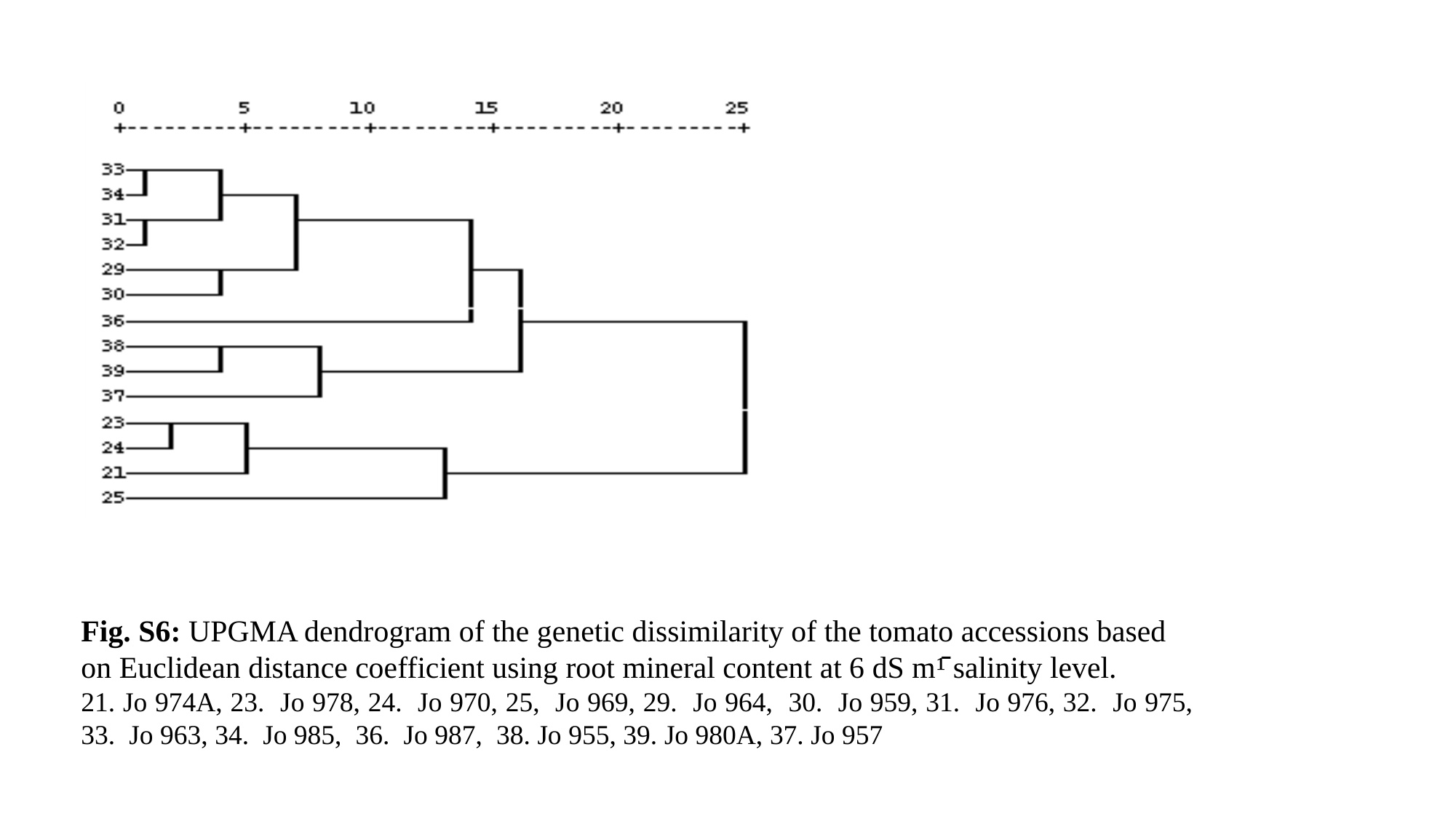

Fig. S6: UPGMA dendrogram of the genetic dissimilarity of the tomato accessions based on Euclidean distance coefficient using root mineral content at 6 dS mֿ¹ salinity level.
21. Jo 974A, 23. Jo 978, 24. Jo 970, 25, Jo 969, 29. Jo 964, 30. Jo 959, 31. Jo 976, 32. Jo 975, 33. Jo 963, 34. Jo 985, 36. Jo 987, 38. Jo 955, 39. Jo 980A, 37. Jo 957
